# W_18_O_49_ Nanowhiskers Decorating SiO_2_ Nanofibers: Lessons from *In Situ* SEM/TEM Growth to Large Scale Synthesis and Fundamental Structural Understanding

**DOI:** 10.1021/acs.cgd.3c01094

**Published:** 2023-12-05

**Authors:** Vojtech Kundrat, Kristyna Bukvisova, Libor Novak, Lukas Prucha, Lothar Houben, Jakub Zalesak, Antonio Vukusic, David Holec, Reshef Tenne, Jiri Pinkas

**Affiliations:** † Department of Molecular Chemistry and Materials Science, Weizmann Institute of Science, Rehovot 7610001, Israel; ‡ Thermo Fisher Scientific, Vlastimila Pecha 12, CZ-62700 Brno, Czech Republic; § Department of Chemistry, Faculty of Science, Masaryk University, Kotlarska 2, CZ-61137 Brno, Czech Republic; ∥ CEITEC BUT, Brno University of Technology, Purkynova 123, CZ-61200 Brno, Czech Republic; ⊥ The Czech Academy of Sciences, Institute of Scientific Instruments, Kralovopolska 147, CZ-61264 Brno, Czech Republic; # Department of Chemistry and Physics of Materials, University of Salzburg, Jakob-Haringer-Str. 2A, A-5020 Salzburg, Austria; ∇ Department of Materials Science, Montanuniversität Leoben, Franz-Josef-Straße 18, A-8700 Leoben, Austria; ¶ Department of Chemical Research Support, Weizmann Institute of Science, Rehovot 7610001, Israel

## Abstract

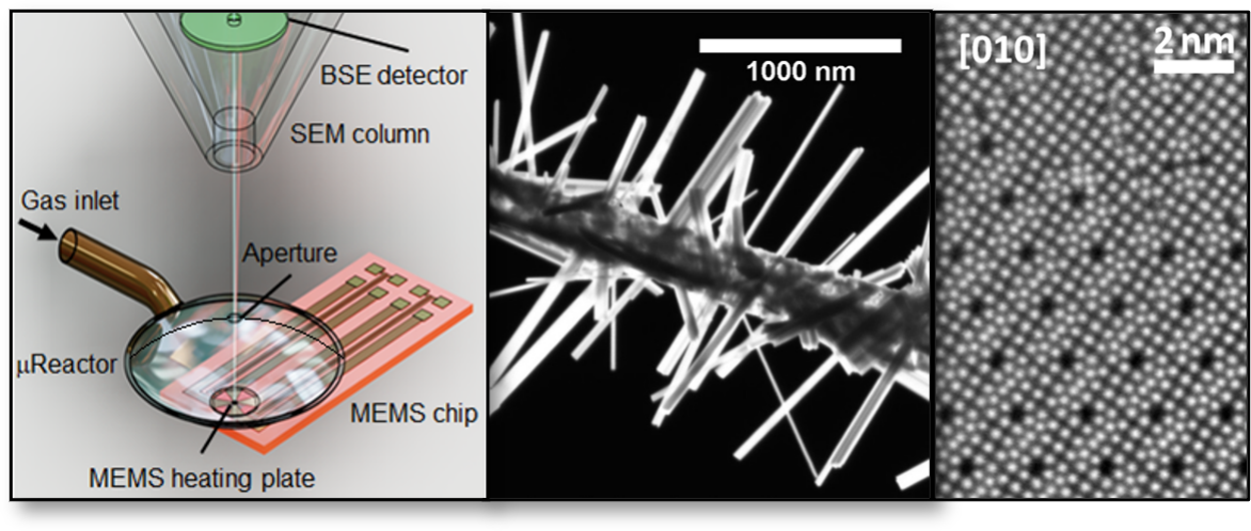

Tungsten suboxide W_18_O_49_ nanowhiskers are a material of great interest due to their potential high-end applications in electronics, near-infrared light shielding, catalysis, and gas sensing. The present study introduces three main approaches for the fundamental understanding of W_18_O_49_ nanowhisker growth and structure. First, W_18_O_49_ nanowhiskers were grown from γ-WO_3_/*a*-SiO_2_ nanofibers *in situ* in a scanning electron microscope (SEM) utilizing a specially designed microreactor (μReactor). It was found that irradiation by the electron beam slows the growth kinetics of the W_18_O_49_ nanowhisker, markedly. Following this, an *in situ* TEM study led to some new fundamental understanding of the growth mode of the crystal shear planes in the W_18_O_49_ nanowhisker and the formation of a domain (bundle) structure. High-resolution scanning transmission electron microscopy analysis of a cross-sectioned W_18_O_49_ nanowhisker revealed the well-documented pentagonal Magnéli columns and hexagonal channel characteristics for this phase. Furthermore, a highly crystalline and oriented domain structure and previously unreported mixed structural arrangement of tungsten oxide polyhedrons were analyzed. The tungsten oxide phases found in the cross section of the W_18_O_49_ nanowhisker were analyzed by nanodiffraction and electron energy loss spectroscopy (EELS), which were discussed and compared in light of theoretical calculations based on the density functional theory method. Finally, the knowledge gained from the *in situ* SEM and TEM experiments was valorized in developing a multigram synthesis of W_18_O_49_/*a*-SiO_2_ urchin-like nanofibers in a flow reactor.

## Abstract 2

W_18_O_49_ nanowhiskers growth from electrospun γ-WO_3_/*a*-SiO_2_ nanofibers is studied by *in situ* SEM and TEM experiments. For the *in situ* SEM, new μReactor technology is fabricated, allowing high-temperature reduction under hydrogen gas (100 Pa). Resulting knowledge from *in situ* experiments was exploited for the multigram synthesis of W_18_O_49_ nanowhiskers decorating *a*-SiO_2_ nanofibers in a flow reactor.

## 1 Introduction

Substoichiometric tungsten oxides WO_3–*x*_, especially nanoscopic forms of W_18_O_49_, are promising materials due to their many exciting properties, resulting in potential applications in various technologies, like electrochromic devices, sensors, etc.^1−5^ W_18_O_49_ exhibits near-infrared absorption,^6,7^ which could be exploited for heat shielding,^8^ heat generation, and water evaporation closely connected to recently demonstrated water desalinization.^9−11^ The tungsten suboxides could also be used in the photoreduction of carbon dioxide,^12^ photocatalysis,^4,5,13,14^ electrocatalysis,^4^ and photoluminescence.^4,5^ The tungsten suboxides’ characteristics, including their distorted inner structure, their highly oxygen-deficient structure, and their nanoscopic size, were examined in many studies.^1−4,15−17^ The structure containing triangular and hexagonal channels allows W_18_O_49_ to act as an anodic material for lithium-ion batteries.^18−21^ Intercalation of lithium ions into the W_18_O_49_ structure was studied for triggered lattice contraction, which results in macroscopic material flexibility and electrochromic color change.^22^ W_18_O_49_ was successfully exploited as a precursor in the large-scale synthesis of WS_2_ nanotubes.^23^ Another feature of this material is its sensory properties for NO_*x*_ and ammonia gases.^24−26^

There are several different approaches for preparing nanoscopic W_18_O_49_ and other tungsten suboxides in nanowhisker or nanofibrous forms that differ from each other in a synthetic method or in a different tungsten-containing precursor. The most frequently used method is based on the solvothermal reaction of WCl_6_^26,27^ or W(CO)_6_^28,29^ with aliphatic alcohols in an autoclave or just at elevated temperature in high boiling point solvents. This route results in uniform nanowhiskers of various lengths and thicknesses. Other possible preparation procedures involve controlled oxidation of metallic tungsten with water vapor^23,30^ or flame synthesis based on the deposition of tungsten oxides on various surfaces by heating a tungsten mesh with a high-temperature heater.^31^ Tungsten disulfide WS_2_ could also be used as a tungsten source for these nanofibers using the high-temperature, low-pressure reactor in the presence of water vapor.^32^ This study was conducted in a chamber of an electron microscope with a heating extension serving as a sample holder. A similar study^33^ has been done based on the oxidation of tungsten filament to substoichiometric tungsten oxide whiskers with characterization and material properties testing performed entirely *in situ* in the electron microscope chamber.

Electrospinning^34−36^ is a versatile and accessible method for the production of submicron and nanoscopic fibers from organic polymers and inorganic compounds with a valuable extension to the industrial production of many high-end materials and products. There are suitable routes for preparing various inorganic oxides, carbides, sulfides, or even metallic nanofibers based on multistep processes involving electrospinning. For electrospinning of the inorganic nanofibers, appropriate solutions were prepared first. Usually, the inorganic precursors containing the desired elements are dissolved with an organic, supporting polymer in a suitable solvent. The prepared solution is electrospun into the so-called green composite nanofiber web. The following step is a high-temperature calcination in air, which removes the organic part obtaining pure inorganic oxide fibers.^37^ Nanofibers containing the W_18_O_49_ phase were prepared in the past by a combination of electrospinning of a suitable substrate and solvothermal synthesis of desired W_18_O_49_ nanowhiskers from WCl_6_. So far, materials based on carbon,^38^ TiO_2,_^39^ and polyacrylonitrile^40^ nanofibers have been exploited as substrates for decoration by W_18_O_49_ nanowhiskers.

Recently, we have described a multigram preparation of tungsten trioxide/amorphous silica (for brevity γ-WO_3_/*a*-SiO_2_) fibers, which served as a precursor material for the preparation of polycrystalline tungsten metal nanofibers^41^ and WS_2_ microfibers.^42^ In both cases, amorphous SiO_2_ acted as a binder of the material nanograins. During the reduction of WO_3_ fibers to metallic tungsten, several reduction processes were observed with varying compositions and morphologies.

To further this study, we have focused in the present work on the growth of the W_18_O_49_ nanowhiskers on the silica fibers using a new technique, i.e., the microreactor (μReactor), which is an *in situ* reactor in the scanning electron microscope (SEM). The reactor includes a microelectromechanical system (MEMS) heating chip (referred to in the text as MEMS chip or MEMS heating chip), which can be transferred to *in situ* transmission electron microscopy (TEM). This reactor was briefly described in a previous study.^32^ Here, the new μReactor and the growth process, i.e., the *in situ* reduction of the γ-WO_3_/*a*-SiO_2_ fibers in SEM and TEM, are described in great detail.

The growth of W_18_O_49_ nanowhiskers from heated and partially oxidized tungsten filament in TEM was described by Hashimoto et al. in 1960.^43^ The mechanism of the process was profoundly studied by Zhang et al.,^44^ who described mainly the mass transport during the oxidative W_18_O_49_ nanowhisker growth from tungsten filament. Here, the reaction was carried out with an environmental transmission electron microscope (ETEM). Alternatively, a direct reduction of tungsten oxide was observed *in situ* in TEM,^45^ similarly in refs (43 and 46). These results show that W_18_O_49_ nanowhiskers grow in an anisotropic fashion involving volatile tungsten oxides from oxidized metallic tungsten or by e-beam reduction of tungsten oxide.

In sharp contrast with the *in situ* experiments described above, the μReactor, described in great detail below, operates under precisely controlled conditions. Therefore, the growth conditions used in the μReactor can be straightforwardly utilized for the optimized growth of W_18_O_49_ nanowhiskers in the low-pressure flow reactor, as shown in the present study.

There are three pillars to the present work:

- Kinetic study of the W_18_O_49_ nanowhisker growth at elevated temperatures using the *in situ* μReactor (consisting of a MEMS heating chip and accessories) within the SEM.
- Following the detailed structural changes occurring during the W_18_O_49_ nanowhisker growth using *in situ* experiments in the TEM, furnished with the same MEMS heating chip previously used in the SEM. Moreover, fundamental insight into the structure of the nanowhiskers and their characteristics are provided by analysis of nanowhisker cross section using advanced microscopy techniques in synergy with theoretical calculations gained through the density functional theory (DFT) method.
- Transfer of knowledge gained from the two previous *in situ* investigations to optimize the reaction conditions in the scaled-up low-pressure tube furnace for the growth of W_18_O_49_ nanowhiskers decorating amorphous silica nanofibers.

## 2 Experimental Section/Methods

### 2.1 General

Polyvinyl alcohol (PVA, Mowiol 18–88) and silicotungstic acid hydrate H_4_SiW_12_O_40_ (HSiW, purum) were purchased from Merck and used as received. Deionized water was used as a solvent. For electrospinning, a Nanospider NS LAB500S instrument (Elmarco, Czech Republic) equipped with a cylindrical electrode with microblades for allocation of the electrical charge and solution droplets was used (shown schematically in Figure S1). Electrospinning preparation of green composite fibers consisting of PVA and HSiW was described in a previous study.^42^ The experimental procedure used here is described in the Supporting Information, Experimental Section.

### 2.2 Calcination of Tungsten Oxide Precursors

The green fibers of PVA and HSiW were calcined in air at 600 °C in a muffle furnace. The furnace was heated within 1 h to the final temperature followed by a 2 h dwell time. After heat treatment, the sample was left to cool spontaneously to ambient temperature. The prepared γ-WO_3_/*a*-SiO_2_ nanofibrous material was analyzed by SEM and X-ray powder diffraction (XRD) (Supporting Information Experimental Part 1).

### 2.3 Characterization

X-ray powder diffraction (XRD) measurements were performed with an Empyrean diffractometer (PanAnalytical) with a Co (λ_Kα_ = 1.79030 Å) X-ray lamp at room temperature. The phase analysis was performed by the Rietveld method; the crystallite size was determined via the Scherrer equation by HighScorePlus 4.0 (PanAnalytical) using the ICSD database.

### 2.4 Scanning Electron Microscopy

The electrospun nanofibrous materials were characterized by SEM with a Versa 3D (Thermo Fisher Scientific) microscope and by scanning transmission electron microscopy (STEM) on an FEI Magellan 400 XHR microscope (Thermo Fisher Scientific). A Helios UC Focused-Ion-Beam (FIB)-SEM system (Thermo Fisher Scientific) was used for the *in situ* SEM experiments in the μReactor and the lamella preparation from a W_18_O_49_ nanowhisker. For the lamella preparation, W_18_O_49_ nanowhiskers were dispersed in isopropyl alcohol and dropcasted on a Si substrate. A Helios 5 FX FIB microscope was used for localization of a specific nanowhisker, micromanipulation and final lamella preparation. A gas injection system (GIS) was utilized for creating of an amorphous carbon protection layer. First, a deposition step was performed by electron-assisted deposition and later ion assisted deposition was used. Chunk thinning and final polishing operations were performed at FIB accelerating voltages ranging from 30 kV to 2 kV and FIB currents ranging from 2 nA to 25 pA. The SEM micrographs were analyzed by the ImageJ software to determine the fiber diameters and the size distribution.

### 2.5 Transmission Electron Microscopy

Samples were drop-cast onto copper grids with a lacey carbon support film after the sample suspension was sonicated in methanol. Energy dispersive X-ray spectroscopy (EDS) data were measured on a Thermo Fisher Scientific Talos F200i equipped with a Bruker Dual-X spectrometer, operated in the STEM regime at a high voltage of 200 kV and beam current of 0.5 nA. Spectra and images were postprocessed by the Velox software.

Electron energy loss spectra (EELS) were measured with a CEFID (Ceos GmbH, Heidelberg, Germany) spectrometer on a double aberration-corrected Themis-Z microscope (Thermo Fisher Scientific Electron Microscopy Solutions, Hillsboro, USA) at an accelerating voltage of 200 kV. The EELS data was recorded in STEM mode at a beam current of 60 pA, a semiconvergence angle of 21 mrad, and a semicollection angle of 60 mrad, using an ELA direct detection camera (Dectris AG, Baden, Switzerland). Radiation damage to the tungsten oxide was minimized by repeated rapid frame scanning in a focus frame while the energy-loss spectra. The fine calibration of the spectrometer’s energy scale was performed against standards (Si, a-Al_2_O_3_, NiO, BN, C) and respective XANES reference data reported in the NIMS database.

Scanning electron diffraction data were recorded with the CEFID on the ELA detector in zero-loss filtered mode. An electron probe with a convergence angle of 0.2 mrad was adjusted in STEM microprobe mode and further defocused to reduce the electron flux by enlarging the probe size to about 10 nm. A primary beam current of less than 5 pA was used.

### 2.6 Density Functional Theory

The description of density functional theory calculations of the oxygen K core-loss EELS spectrum is given in the Supporting Information Experimental Part 2.

### 2.7 *In Situ* Reaction in SEM on the MEMS Chip – The μReactor Technology

**Figure 1:**
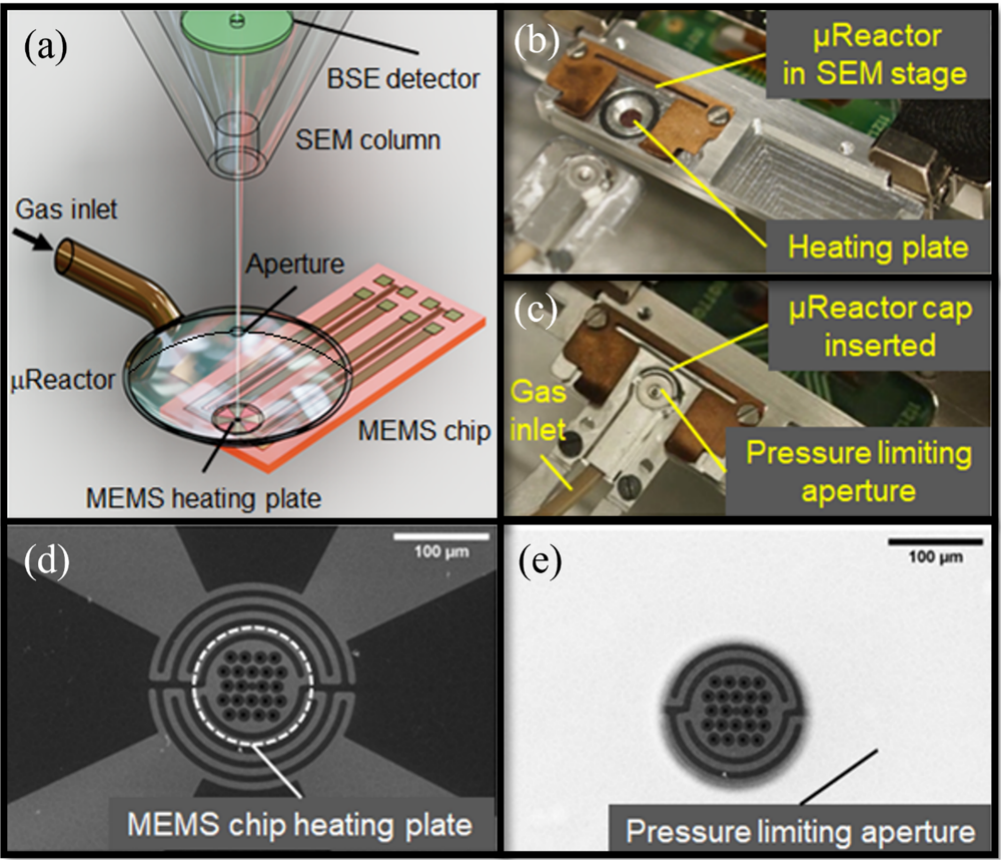
(a) Scheme of the μReactor in the SEM. (b) Photograph of the μReactor with the retracted cap. (c) Photograph of the μReactor closed by the inserted cap. (d) SEM image of the MEMS heating plate. (e) SEM image of the MEMS heating plate with the closed cap. The reaction volume is open during sample placement on the heating plate (b, d) and sample manipulation by the EasyLift needle. When a sample is in place and aligned under the electron beam (e-beam), the manipulator needle is retracted, and the cap of the μReactor is inserted (c). The reaction volume is thus closed and sealed. Overpressure inside the reactor (up to 500 Pa) is assured by the pressure-limiting aperture in the cap viewed by the SEM from the top (white area in (e)). Note that the SEM chamber remains under high vacuum conditions. *In situ* SEM imaging is possible through the hole in the aperture (e). The gas inlet is incorporated into the cap. Gas escapes from the μReactor mainly through the aperture. The reactor can be opened/closed inside the SEM chamber without a need for chamber venting, which ensures clean sample preparation (by FIB) and sample positioning (by the manipulator EasyLift needle) without exposure to air. The sample deposited on the heating segment of the MEMS chip reacts with the admitted gas or under vacuum at elevated temperatures.

The μReactor is based on an SEM stage containing a MEMS chip with a microheating plate covered by a cap containing a pressure-limiting aperture and a gas inlet (Figure 1). By covering the stage with the cap, the μReactor body is closed, permitting locally increased gas pressure (up to 500 Pa) of various inlet gases. A sample deposited on the microheating plate in the μReactor is heated by the Joule effect (up to 1200 °C with a maximal heating rate of 4 × 10^4^ K s^–1^). Very fast sample heating and cooling allow for a precise control of the reaction setup and kinetic measurements. The heated specimen eventually reacts with a gaseous atmosphere. The reaction is continuously monitored by using the signal of backscattered electrons (BSE) or secondary electrons (SE), which are detected inside the SEM column. Obviously, the electrons of the beam can intervene in the reaction directly and indirectly by heating the specimen.

A Helios UC FIB-SEM system (Thermo Fisher Scientific) with the μReactor was used for the *in situ* SEM imaging of the growth of nanowhiskers (shown schematically in Figure 1a).^41^ The μReactor is described in detail in Figure 1b–e. It allows forming of a local overpressure of the processing gas (up to 500 Pa) when placed inside an SEM chamber, while the SEM is operated in a standard high-vacuum mode (with a chamber pressure of ⟨10^–2^ Pa). The sample heating inside the μReactor is provided by an MEMS-based microheating plate^54^ allowing maximum temperature up to 1200 °C (Figure 1d). The temperature of the heating plate is controlled from the SEM user interface.^55^

The processed samples were placed manually on the microheating plate (Figure 1d) by tweezers and shifted to the desired position using an EasyLift manipulator needle. Once the sample was placed on the heating plate and aligned under the electron beam, the cap of the μReactor was inserted to seal the reaction volume (Figure 1c,e). *In situ* imaging was done by an in-lens (TLD) detector collecting signals of secondary (SE) and backscattered (BSE) electrons passing through an aperture hole in the reactor cap (Figure 1e). Pure hydrogen was admitted into the reaction volume through a capillary connecting the μReactor cap (Figure 1e) with a gas feedthrough on the SEM chamber. The inlet flow rate was controlled by a mass flow controller placed outside the SEM chamber between the hydrogen cylinder and the gas feedthrough of the SEM chamber.

The temperature of the heating plate was calculated from the resistance of the heated wire. The pressure inside the reaction volume was estimated from the heating power, which depends on the pressure of the surrounding gas, employing the Pirani gauge principle. The advantage of this approach is that both the temperature and pressure can be measured simultaneously and directly in the vicinity of the sample without the need for a bulky thermocouple or a large volume pressure gauge connected to the μReactor.

In another embodiment of the experiment (experiment No. 2, see Supporting Information Discussion 2 and its visualization in Video 2), the precursor (γ-WO_3_/*a*-SiO_2_ nanofibers) was heated in the μReactor without being exposed to hydrogen gas by heating the specimen under high vacuum. In this case, the cap of the reactor was retracted, and the reaction volume was opened to the SEM chamber, as shown in Figure 1b,d.

Generally speaking, there are three main controllable variables in the reaction: temperature (heating profile), gas pressure, and time. Both temperature and pressure can be continually adjusted during the *in situ* experiment and their values are continuously monitored based on the heating circuit resistance and power.

Monitoring the growth of the nanowhiskers *in situ* by the SEM permits immediate cutoff of the heating, which stops the reaction instantaneously, anytime. The duration of the reaction is therefore fully variable based on the SEM observation.

### 2.8 Heat Treatment – A Partial Reduction in the SEM μReactor

To prepare a sample for heat treatment in the SEM μReactor, the calcined fibers in the form of flakes were placed on the MEMS heating chip and rubbed on the surface by a micromanipulator. Using this method, several individual nanofibers were deposited on the heating area in the center of the MEMS chip. For the acquisition, the acceleration voltage was set to 10 kV and the current to 0.4 nA. After preparing of the sample, heating was set up to 700 °C (heating rate 2 K s^–1^). Once the sample reached this temperature, the SEM imaging was brought to focus again, and the temperature was increased to 800 °C or more. The raster scan speed was variably set to 20.5 or 41 s per image. After the reaction ended, the magnification was reduced to see the whole set of fibers on the MEMS chip.

Of the several experiments carried out with the γ-WO_3_/*a*-SiO_2_ nanofibers in the SEM μReactor, two are described in greater detail. Their reaction parameters and SEM acquisition settings are listed in Table 1. Experiment No. 2 is discussed in the Supporting Information Discussion 2.

**Table 1:** Processing Conditions of Experiments Conducted in the μReactor

exp./video No.	temperature [°C]	H_2_ press./vac. [Pa]	SEM image frame time [s]
1	800	100	20.5
2	900	1.10^–4^ (vacuum)	41

Video S1 was formed by collating a sequence of SEM images from experiment No. 1 at a rate of 3 frames per second. Individual frames were acquired at SEM imaging every 20.5 s. One second in the video corresponds to approximately 60 s in real reaction time. Similarly, Video S2 (from experiment No. 2) was constructed at a rate of 1 frame per second every 41 s. Therefore, one second in the video represents 41 s in real reaction time.

### 2.9 *In Situ* Reaction in the TEM using the MEMS Chip

Originally, the MEMS chip (Figure S6a) was fabricated and used for the *in situ* TEM experiments.^54^ The TEM observation was conducted through a perforated, thin, amorphous silicon nitride membrane attached to the heating plate (Figure 1d and Figure S6b). The MEMS chip with the deposited sample was placed into the Thermo Fisher Scientific NanoEx-i/v heating and biasing holder for *in situ* STEM imaging and elemental analysis (EDS) at elevated temperatures (Figure S6c). The holder is similar to the standard single-tilt holder used for analysis of TEM grids, however, with a rectangular fitting for the MEMS chip and with contacts for an external power supply. *In situ* experiments were performed on a Thermo Fisher Scientific Talos F200i microscope equipped with a Bruker Dual-X (EDS) spectrometer, operated in the TEM regime at a high voltage of 80 kV and beam current of 1 nA. The setup allows continual measurements with real-time video output of the process. The *in situ* annealing was observed at 820 °C. The *in situ* TEM experiment was acquired as real-time Video S3 and Video S4.

### 2.10 Heat Treatment – Partial Reduction in a Tube Furnace

The same conditions used for obtaining the fibers *in situ* in the SEM experiments were also tested in a custom-made tube furnace (Figure S7a). This furnace could optionally operate under vacuum or hydrogen atmosphere with a controllable pressure. The furnace allowed also shock heat treatment by moving the furnace, fixed on a rail toward the sample boat.

The prepared γ-WO_3_/*a*-SiO_2_ nanofibers (calcined at 600 °C) were placed in an alumina combustion boat and were covered with another one to prevent the material from spilling out during evacuation (see Figure S7b). The boat with the sample was placed into a quartz tube and initially evacuated to reach a high vacuum (1 × 10^–4^ Pa) followed by setting up a partial hydrogen atmosphere by allowing hydrogen from the gas cylinder into the quartz tube and partially closing the valve into the vacuum pump system. By careful optimization of the inlet and outlet valves, the pressure was set to a constant value of 100 ± 5 Pa. The furnace was preheated at 800 °C and after reaching the desired temperature, and the furnace was moved to the region of the quartz tube with the sample. In this way, shock calcination was performed for 1 h, followed by spontaneous cooling to ambient temperature, venting, and collecting the resulting material for structural and chemical evaluation.

## 3 Results and Discussion

### 3.1 Preparation of the Precursor (γ-WO_3_/*a-*SiO_2_) Nanofibers and their Characterization

The electrospinning setup (Nanospider) suitable for the multigram synthesis of W and WS_2_ fibers was described elsewhere.^42^ In the past, silicotungstic acid (HSiW) was used as a useful tungsten oxide precursor for electrospinning. The fabricated green nanofibers were calcined at 600 °C. Careful analysis showed that they consist of WO_3_ grains attached to an amorphous silica phase (for brevity, they are named γ-WO_3_/*a*-SiO_2_). The mean diameter of the γ-WO_3_/*a*-SiO_2_ fibers was 225 ± 88 nm. The preparation process of the precursor and material characterization are presented in the Supporting Information, Experimental Part 1.

### 3.2 *In Situ* Reaction in the μReactor within SEM and Optimization of the Reaction Conditions

Tungsten trioxide undergoes reduction at elevated temperatures under a vacuum or in a hydrogen atmosphere, releasing oxygen or water vapor, respectively. The reduction pathway proceeds through various stable suboxide phases (e.g., W_20_O_58_ and W_18_O_49_) to WO_2_ and finally to elemental tungsten. WO_3_ consists of a three-dimensional array of corner-shared [WO_6_] octahedra. The different tungsten suboxide phases formed during the reaction have in common a rearrangement of the corner-shared [WO_6_] octahedra into structures with shared edges. This transformation induces the formation of aligned crystallographic shear (CS) planes. The formation of the CS planes eventually leads to a highly anisotropic growth like that of the W_18_O_49_ nanowhiskers (monoclinic unit cell; space group *P*2/*m* with *a* = 1.83, *b* = 0.38, and *c* = 1.4 nm). The nanowhiskers have the tendency to form bundles during growth,^43^ which adversely affects their further processing into functional electronic devices or converting them into WS_2_ nanotubes of high crystalline order.

As already stated, W_18_O_49_ nanowhisker growth was studied by multiple approaches. On the one hand, the reaction mechanism and the kinetics could be revealed by *in situ* TEM experiments.^43,44^ Alternatively, experiments carried out here using *in situ* SEM μReactor and under entirely different conditions permitted the visualization of the overall process as a function of the growth parameters. Here, the precise heating profile available by the MEMS chip and the hydrogen pressure tuning within the bell-jar-shaped μReactor made the tool ideal for optimization of the reduction reaction. Figure 1 presents the μReactor with the MEMS chip within the SEM used in the present study. Full details of the μReactor with its MEMS chip is provided in the Experimental Section. It is important to realize that the pressure inside the μReactor is ∼500 Pa (5 Torr), while the vacuum in the SEM chamber is 10^–2^ Pa (10^–4^ Torr). The following discussion describes the optimization of the W_18_O_49_ nanowhiskers growth from the γ-WO_3_/*a*-SiO_2_ nanofibers in a hydrogen atmosphere and under vacuum at elevated temperatures (up to 900 °C) using the μReactor within the SEM. Of the several experiments carried out in the μReactor within the SEM, two are described in detail (Table 1 in the Experimental Section). The two experiments differed in their reaction temperature and hydrogen pressure. The goal was to study the whole reduction process while observing the morphological changes.

### 3.3 Experiment No. 1 - Partial Reduction of γ*-*WO_3_/*a*-SiO_2_ Nanofibers at 800 °C in 100 Pa H_2_ Atmosphere

**Figure 2:**
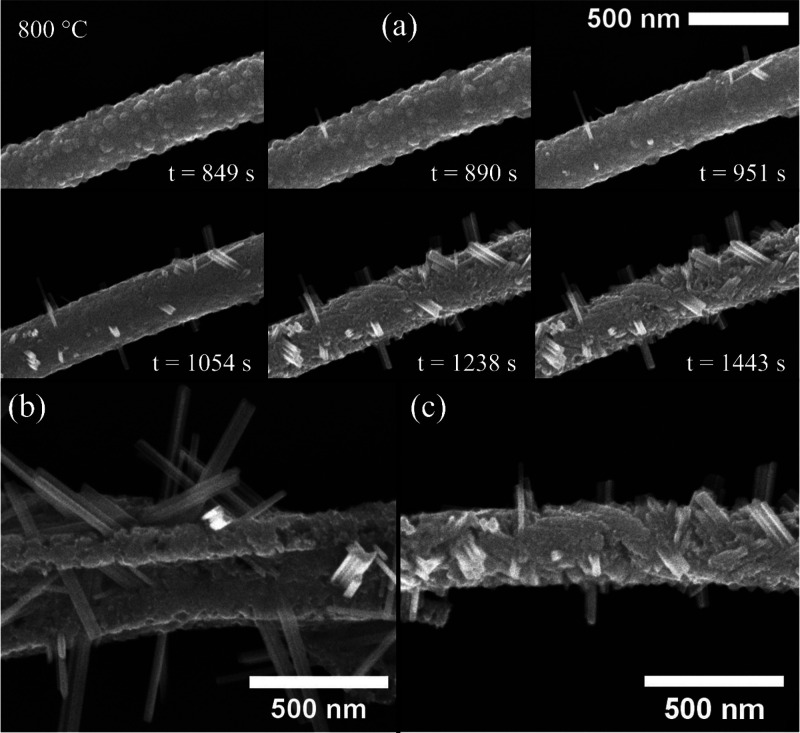
(a) SEM image of the γ-WO_3_/*a*-SiO_2_ nanofibers heat treated in the μReactor within the SEM (maximum temperature 800 °C, 100 Pa of H_2_). (b) SEM image of area not irradiated by the e-beam. (c) SEM image of the area irradiated by the e-beam. Individual images in (a) were selected from those used to produce Video S1. Once heated to 800 °C (*t*_reaction_ = 849 s), the nanowhiskers started growing. Simultaneously with that, the protrusions on the nanofiber surface (particulates) disappeared and the fiber surface smoothed (*t*_reaction_ = 951 s). The growth of nanowhiskers in the first phase of the reaction (*t*_reaction_ ⟨ 1054 s) was followed by the continuous emergence of new neighboring nanowhiskers (approximately *t*_reaction_ = 1053–1238 s). At the final stage of the reaction, the fiber surface was decorated by bundles of protruding nanowhiskers and coarse particles (*t*_reaction_ ≥ 1443 s) all around. The image of the nonirradiated area in (b) was obtained (after the reaction termination) for morphological comparison with the e-beam-irradiated nanowhisker in (c). The mean lengths of the e-beam-exposed and nonirradiated nanowhiskers were 92 ± 36 and 391 ± 134 nm, respectively.

In this experiment, the growth of W_18_O_49_ nanowhiskers on the surface of γ-WO_3_/*a*-SiO_2_ nanofibers in the μReactor was undertaken. The W_18_O_49_ nanowhiskers grew on the entire surface protruding outside the γ-WO_3_/*a*-SiO_2_ nanofiber. The electron beam (e-beam) of the SEM was focused on an individual nanofiber, which is shown in Video S1 with selected frames presented in Figure 2a. The rest of the nanofibers in the μReactor were reacted without being visualized *in situ*, i.e., without being exposed to the e-beam, which apparently had a substantial influence on the growth mode of the nanowhiskers (*vide infra*). The nanowhisker growth on the (e-beam) irradiated nanofiber started at 800 °C (Figure 2a, *t*_reaction_ = 849 s), which was accompanied by smoothening of the γ-WO_3_/*a*-SiO_2_ fiber’s surface (Figure 2a, *t*_reaction_ = 951 s). Presumably, the even surface formation was caused by mass transport of the tungsten oxide from the γ-WO_3_/*a*-SiO_2_ fiber’s body into the W_18_O_49_ nanowhiskers. Consequently, nanowhiskers grown early on seeded formation of neighboring whiskers further during the reaction, thus collectively generating bundles (Figure 2a, *t*_reaction_ ⟨ 1054 s) on the surface contour of the γ-WO_3_/*a*-SiO_2_ nanofiber. The emergence of W_18_O_49_ nanowhiskers into bundles was reported previously.^43^ At this point, the original nanofiber was partially covered with multiple W_18_O_49_ nanowhiskers forming a bundle, which protruded outside. In addition, surface coarsening of the e-beam irradiated nanofiber was observed (see the arrow in Figure 2a at *t*_reaction_ = 1443 s). The surface coarsening of the nanofibers occurred on scars in the vicinity of the nanowhiskers. The nanowhiskers in the final stage of the reaction shrunk into bundles.

**Figure 3:**
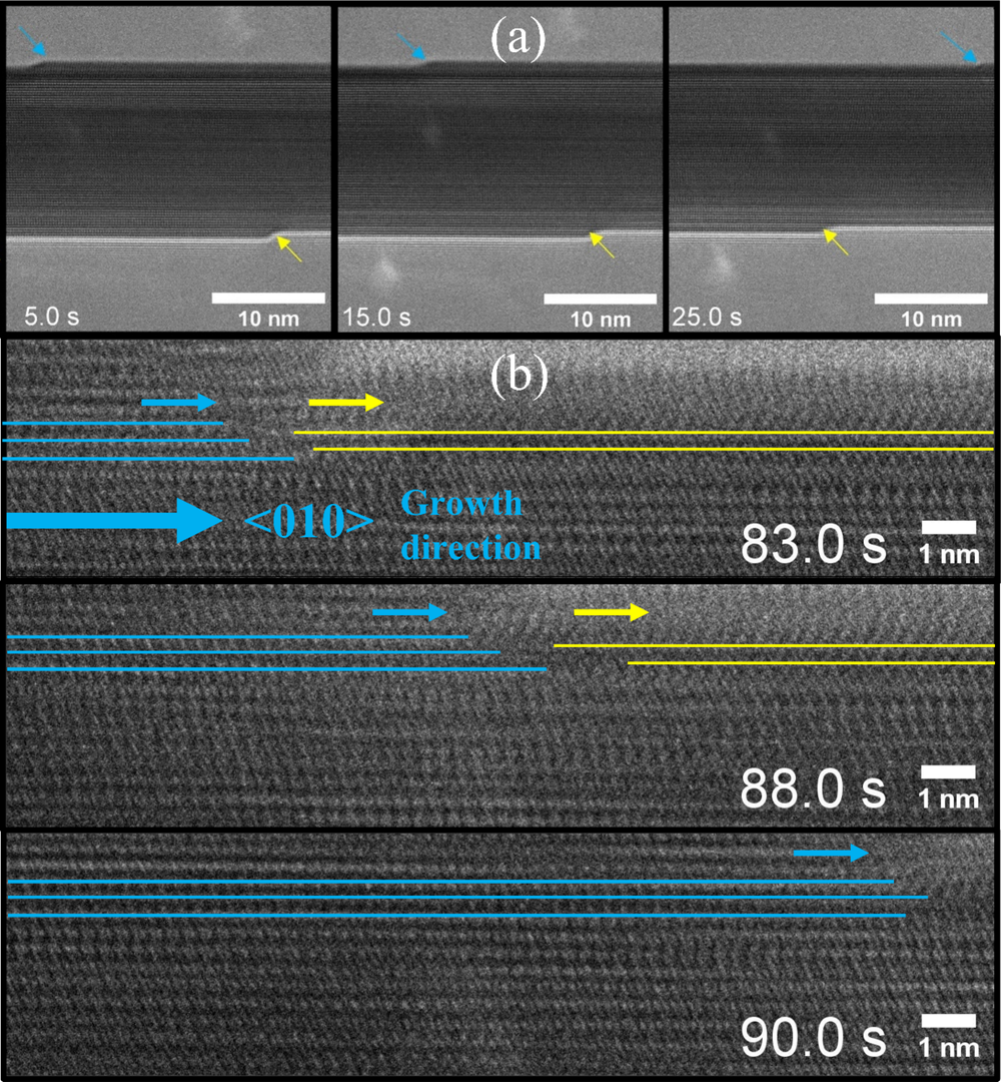
*In situ* TEM images of shear plane translations along the ⟨010⟩ axis of the W_18_O_49_ nanowhisker. (a) The process starts with the tips of the distinct shear planes positioned at the edges of the TEM image (*t* = 0 s in Video S5). Gradually, the shear plane tips (marked by the yellow and blue arrows and tilted according to the direction of translation) move with time toward the opposite edges (*t* = 5–25 s). Apparently, the ″blue″ shear plane progresses twice as fast as the ″yellow″ one. The possible explanation is in the local temperature difference or the angling toward the volatile WO_2_(OH)_2_ source where the ″yellow″ slower shear plane has higher oxide supply. Therefore, the evaporation of the ″yellow″ shear plane is slower. (b) Detailed *in situ* TEM images of a growing nanowhisker shown in (a) later during the growth. The blue-marked shear planes emerge during bundle growth. Yellow lines represent already-formed W_18_O_49_ shear planes, which are unaligned with the newly forming ones (blue). By merging the blue shear planes with the yellow ones, we formed a new aligned bundle structure (*t* = 90 s).

Figure 2b shows a fiber that reacted in the μReactor free of e-beam irradiation simultaneously with the irradiated one. The length of the nanowhiskers of the irradiated nanofiber (Figure 2c) are only a quarter of those which grew in the nonirradiated zone (Figure 2b). Statistical analysis of the nanowhiskers’ length of the e-beam irradiated and nonirradiated shows a factor of four difference between the length of the two families (391 ± 134 nm for the nonirradiated and 92 ± 36 nm for the irradiated W_18_O_49_ nanowhiskers). This intriguing finding is attributed to the interaction of the e-beam with the growing nanowhiskers. On the one hand, the e-beam has a strong chemical reducing nature, but on the other hand, the heating effect of the e-beam cannot be absolutely excluded. Indeed, the vapor pressure of tungsten dioxide, which may have been formed, is appreciably smaller than that of WO_3_ at elevated temperatures,^47^ which may explain the lower growth rate of the e-beam irradiated W_18_O_49_ nanowhiskers.

**Figure 4:**
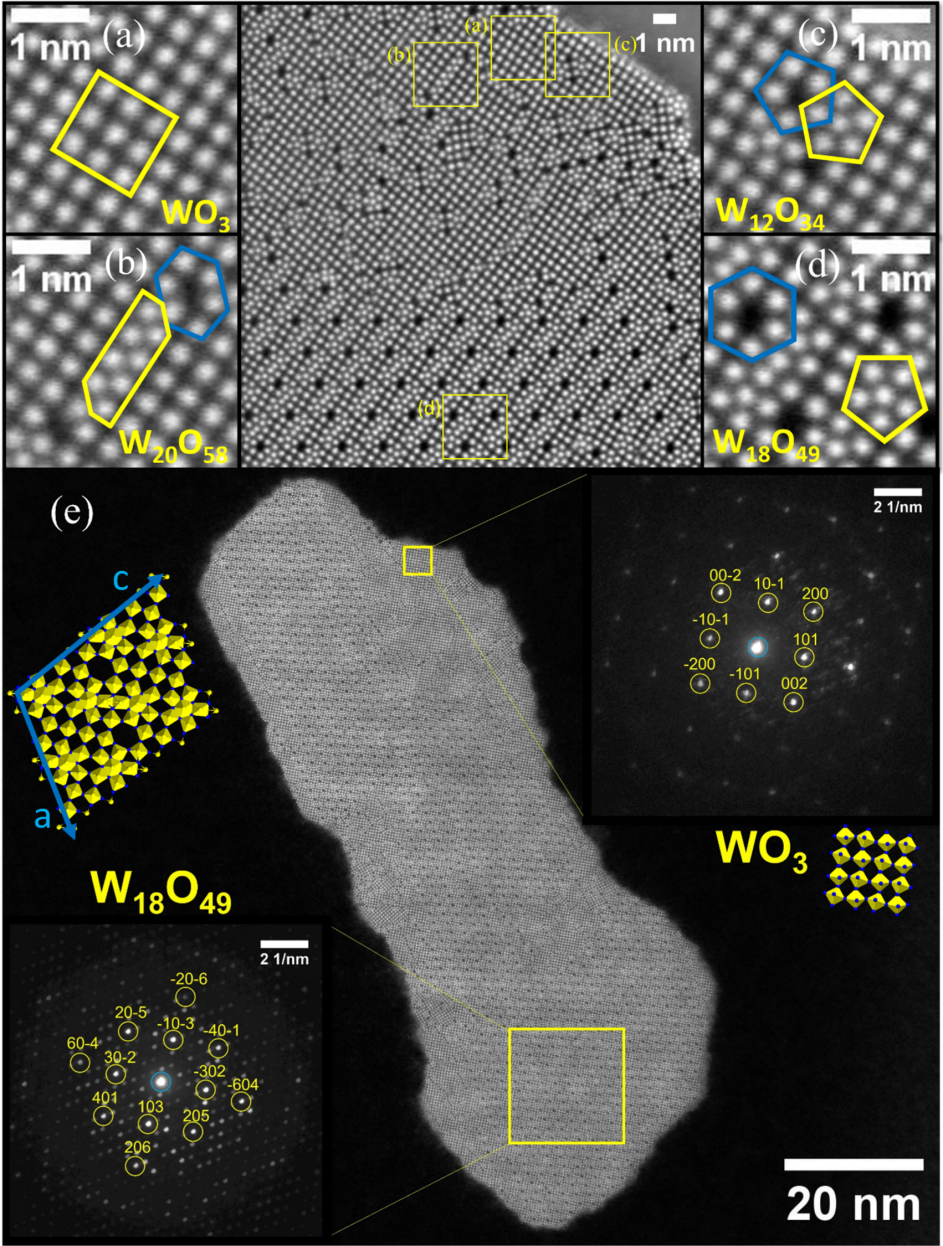
HRSTEM-HAADF measurement of cross-sectioned W_18_O_49_ nanowhiskers. The center of the upper figure shows a typical pattern of the lamella, which is fully displayed in Figure S13A. There are two morphologically different areas: the upper part of the center figure consists of mixture of various structural motifs resembling several known W-O phases. Considerable part of the nanowhisker cross section is formed by array of [WO_6_] octahedra assembled into a WO_3_ lattice seen in (a). Closely stacked arrays of [WO_6_] octahedra are forming a similar lattice to W_20_O_58_ (b). Two pairs of pentagonal columns and pentagonal channels are typical for W_12_O_34_ lattice^15^ and are isolated as square-like motifs in the WO_3_ phase observed in (c). The lower part of the center figure is a pure lattice of the W_18_O_49_ with a typical pattern consisting of pentagonal columns (yellow pentagon in part d) and hexagonal channels (marked blue in d). (e) HRSTEM-HAADF measurement and associated nanodiffractions of selected phases W_18_O_49_ and WO_3_ on the nanowhisker cross section.

**Figure 5:**
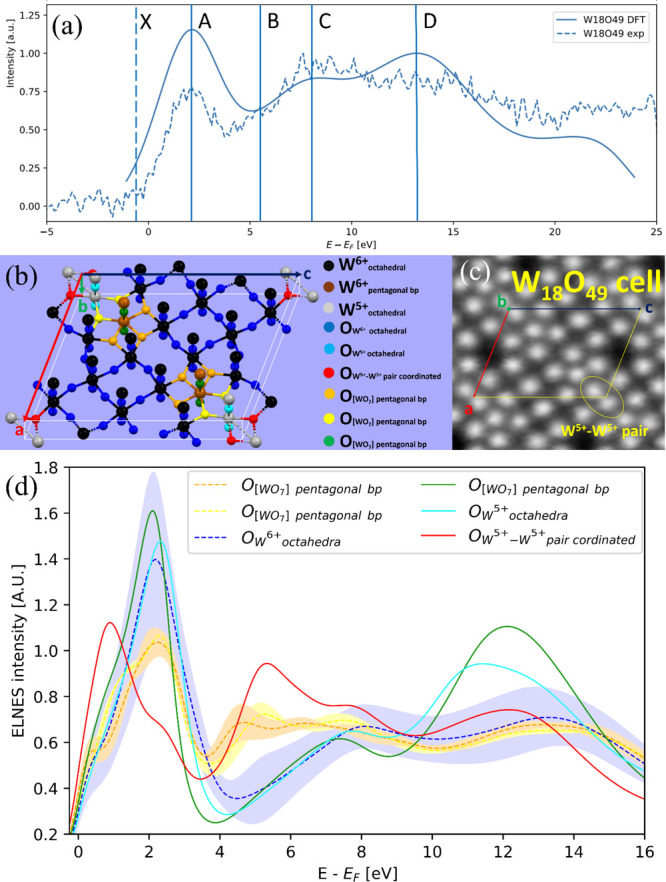
(a) Comparison of the O K-edge ELNES responses from the experiment and the DFT calculations. The first maximum (A, ∼2 eV) in the experimental spectra is at 531.9 eV. The overall offset of the DFT spectra was shifted to match this first experimental and simulated maximum in the spectra. The positions of the peaks on the energy axis at ∼2 (A), ∼8 (C), and ∼12.5 eV (D) agree well between experiment and DFT calculations, although their respective relative ratios of peak intensities differ somewhat. The DFT spectrum overestimates the (A) peak intensity by ∼53% and the (D) peak intensity by ∼26%, while the (C) peak is underestimated by ∼11%. (b) W_18_O_49_ (*P*2/*m*) conventional unit cell with projection on the (010) crystallographic plane (slightly tilted). The applied color coding describes different chemical environments of the constituent O and W atoms. The “bp” in the legend stands for “bipyramide”. (c) TEM bright-field micrograph of the W_18_O_49_ with the marked unit cell in the (010) projection. The O–W^5+^–W^5+^ polyhedra can be seen residing at vertices of the parallelogram projected from the unit cell (encircled by a yellow oval). (d) Dependence of the DFT calculated ELNES response on the different oxygen local environments. The dashed lines represent partial spectra that are averaged by the number of constituent O atoms in the group, while the surrounding same-color “bands” the respective standard deviation of the group. The full lines represent spectra obtained from individual atoms.

Following experiment No. 1, a kinetic study of the growth of WO_3-x_ nanowhiskers (analyzed later as the W_18_O_49_ phase, see Figure 5) on the surface of the e-beam-irradiated γ-WO_3_/*a*-SiO_2_ nanofiber in the μReactor within the SEM was undertaken. For a detailed discussion of the kinetic analysis, see Supporting Information Discussion 1. Experiment No. 2, dedicated to the partial reduction of γ-WO_3_/*a*-SiO_2_ nanofibers at 900 °C under vacuum (10^–3^ Pa, i.e., 10^–5^ Torr), is described in detail in Supporting Information Discussion 2 and visualized in Video S2.

From the carried out experiments, it is evident that the μReactor offers significant advantages for *in situ* studies of heterogeneous reactions and vacuum annealing. Its small volume facilitates clear observations under pressure, which enhances safety, preserves the microscope’s technical condition, and optimizes gas consumption. Chemically speaking, the reductive growth of W_18_O_49_ nanowhiskers is more efficient when hydrogen is used as a reducing agent. Compared to vacuum annealing, the nanowhiskers exhibit a higher aspect ratio, and the reaction allows for more controlled nanofibrous morphology. In both scenarios described above, the W_18_O_49_ nanowhiskers formed bundled structures that protruded from the nanofibrous backbone. In the follow-up experiment, the W_18_O_49_ nanowhiskers were produced and visualized via *in situ* W_18_O_49_ growth experiments in the TEM using a MEMS chip (NanoEx-i/v) for sample heating and direct observation through a silicon nitride membrane.

### 3.4 *In Situ* Reaction in the TEM Using the MEMS (NanoEx-i/v) Heating Chip

The shear plane structure of tungsten suboxides is well-documented.^43,48^ The rearrangement of the corner-shared [WO_6_] octahedra array in WO_3_ (Figure S10a) produces uniform crystal planes with edge-sharing [WO_6_] octahedra. In the case of the W_18_O_49_ nanowhiskers, a unique pattern of pentagonal columns consisting of a [WO_7_] central cluster is surrounded by five [WO_6_] octahedra forming hexagonal channels (Figure S10b).^15,48^

Experiment No. 2 in the μReactor showed the growth of W_18_O_49_ nanowhiskers from the γ-WO_3_/*a*-SiO_2_ nanofibers under vacuum (1.10^–4^ Pa) at 900 °C (see Supporting Information Discussion 2). The same MEMS chip used in the μReactor can also be used for *in situ* TEM experiments utilizing the NanoEx-i/v holder. Therefore, the *in situ* TEM (under high vacuum 1.10^–5^ Pa) allows repeating the reaction under conditions similar to the ones in the μReactor (exp. No. 2). Notably, the growth of the W_18_O_49_ nanowhiskers from the γ-WO_3_/*a*-SiO_2_ nanofibers in the *in situ* TEM experiments was already observed at 820 °C compared to 900 °C as in the *in situ* SEM experiments. The cause of the temperature difference between the two *in situ* SEM and TEM experiments can be presumably attributed to the huge pressure difference in the growth microenvironment (10^2^ Pa in the SEM compared to 10^–3^ Pa in the TEM). The 1 order of magnitude difference in vacuum (10^–2^ in the SEM vs 10^–3^ Pa in the TEM) could also influence the reaction kinetics as well as the energy of the e-beam. The SEM and TEM e-beams were accelerated to 10 and 80 kV, respectively.

The higher resolution of the *in situ* TEM experiment revealed the progress of the shear plane in the growing lattice. The growth is visualized in Video S3 and its selected frames in Figures S11. The recorded growth rate of nanowhiskers was 9 nm s^–1^ in the crystal direction ⟨010⟩. Compared to the reaction in the μReactor within the SEM, the growth rate was more than six times faster for the *in situ* TEM than for the SEM experiments (9 vs 1.45 nm s^–1^). In Video S4 and Figure S12, a startling phenomenon is shown, i.e., the shear planes progress along both the ⟨010⟩ and the opposite ⟨0–10⟩ directions. Thus, the shear plane marked in a yellow rectangle (Figure S12, *t* = 27–45 s) moves from the base of the nanowhisker toward its tip (from right to left). On the other hand, the red-marked shear plane gradually proceeds to the nanowhisker base (from left to right) as seen in *t* = 33–51 s. While both translations take place simultaneously, another visible shear plane, marked by the blue rectangle, is stationary.

The shift of the shear plane was observed *in situ* in TEM on a W_18_O_49_ nanowhisker at 820 °C. It is shown here in higher magnification (×1.05 M) in Video S5 consisting of a sequence of TEM images. Figure 3a is a selected sequence of cropped and rotated images used for the construction of Video S5 (separately shown as Video S5, which is cropped for clarity from the full size Video S7). Distinct shear planes on the surface of a W_18_O_49_ nanowhisker (marked with yellow and blue arrows in Figure 3a) translate toward each other in time. The shear plane marked with a blue arrow translates its tip from the left side toward the right. Simultaneously, the position of the yellow-marked one shifts in the opposite direction. The apparent shear plane shifting observed in Figures 3a and Figure S12 is presumably associated with evaporation and redepositing [WO_6_] octahedra. This redeposition appears to occur predominantly on the tip of the shear planes along the ⟨010⟩ axis of the W_18_O_49_ nanowhisker. Notably, this phenomenon is independent of different shear planes forming in the core of the same nanowhisker. Observation of multiple shear planes (in Figures 3a and S12) in the tungsten suboxide W_18_O_49_ nanowhiskers reveals a bundle-like structure even on the ultrafine level of structural order, i.e., the nanowhisker is not made of a single crystalline domain. Later in the reaction, the nanowhisker went through a series of multiple shear processes that transformed the observed W_18_O_49_ nanowhisker into a bundle structure. This transformation is shown in Video S5 (*t* = 68+ s) and in selected cropped and rotated sequential frames in Figure 3b. Here, a series of new shear planes on the rim of the W_18_O_49_ nanowhisker emerge (see blue lines in Figure 3b) running forward and pushing back existing (yellow marked) W_18_O_49_ shear planes along the ⟨010⟩ direction. Possibly, the blue-marked shear planes forced the yellow ones to shift their position perpendicularly to the growth direction. A fully aligned bundle structure was observed at 90 s in Figure 3b. The reactive growth continued as shown in Video S6.

*In situ* observations revealed discrepancies and misalignments in the shear planes during growth, suggesting the complex structure of the W_18_O_49_ nanowhiskers. As a result, a cross-sectioned lamella of a W_18_O_49_ nanowhisker was produced using a focused ion beam lift-out procedure. This cross section underwent analysis with high-resolution scanning transmission electron microscopy in high-angle annular dark field (HRSTEM-HAADF) mode, as displayed in Figure 4a–d and Figure S13 (which offers a comprehensive view of the lamella). Notably, the nanowhisker’s structure was heterogeneous, encompassing multiple phases.

Previous studies have examined the bundled structure of W_18_O_49_ nanowhiskers with diameters exceeding 80 nm, noting that the space between individual domains was amorphous.^48^ However, in contrast to these findings,^48^ every region of the nanowhisker cross section presented in this study (as seen in Figure 4a–d and Figure S13) was distinctly crystalline and consistently oriented along the [010] axis. This cross section (Figure 4) predominantly features the W_18_O_49_ structure, interwoven with other domains constituted of related W-O phases, specifically the structural motifs of fully oxidized WO_3_ (Figure 4a), W_20_O_58_ (Figure 4b), and W_12_O_34_ (Figure 4c).^49^

The W_18_O_49_ lattice (Figure 4d) embodies two quintessential structural motifs: hexagonal channels formed by six [WO_6_] octahedra and pentagonal columns assembled from a central pentagonal bipyramidal polyhedron [WO_7_], encircled by five [WO_6_] octahedra.^15,49^ These motifs in Figure 4d are highlighted with a blue hexagon and a yellow pentagon, respectively. The emergence of the hexagonal channel can be perceived as compensation for the lattice distortion. This rearrangement stems from the formation of pentagonal column through the removal of oxygen from the WO_3_ lattice. This structural rearrangement produces a relatively stable phase compared with other Mágneli tungsten suboxide phases. Importantly, two edge-linked pentagonal columns^15^ found in the W_18_O_49_ are the structural element motif containing the tungsten in the oxidation state 5+. Counter intuitively, the W^5+^ are not the ones in the pentagonal bipyramidal coordination but the central atoms in octahedra sharing edges.^50,51^ These W^5+^–W^5+^ pairs are responsible for unique absorption properties in the near-infrared regions and other electronic features, like bipolarons.^50,51^

Next to the W_18_O_49_ phase, a significant portion of the analyzed section is composed of a fully oxidized array of [WO_6_] octahedra, forming a WO_3_ lattice (Figure 4a). Between these WO_3_ regions, other structural motifs are observed, notably a lattice resembling the W_20_O_58_ phase (Figure 4b).^49^ In analogy to the W_20_O_58_ lattice, which consists of a repeating unit of three closely packed pairs of [WO_6_] octahedra and a distorted hexagonal channel, the observed motif features an extended pair line by an additional octahedral pair (indicated by a yellow stretched irregular hexagon). The shear plane stacking is counterbalanced by pairs of distorted hexagons (marked in blue), which are also characteristic of the W_20_O_58_ phase. Such W_20_O_58_ resembling a structural motif is present in various places over the analyzed cross section differing by size and orientation. Intriguingly, another arrangement of pentagonal columns was identified (Figure 4c), adhering to the structure of the W_12_O_34_ suboxide.^49^ Two pentagonal columns (marked in yellow) are diamond-linked,^15^ forming a square-like region that is compensated by two pentagonal channels (marked in blue) composed of five [WO_6_] octahedra. This structural feature is isolated within the WO_3_ lattice as seen in Figure 4c.

Further analysis of the multiphase area in the cross section uncovers segments of other suboxides (e.g., W_5_O_14_) and variations in pentagonal column stacking and various defects, which are not discussed any further in the current work. Notably, the cross-sectional analysis may provide insight into the growth discrepancies observed during the *in situ* nanowhisker growth within TEM (Figure 3). The growing structures, misaligned with previous layers, might have contained different phases, which are observed in Figure 3.

Followingly, another lamella from a different nanowhisker was analyzed locally by electron nanodiffraction (Figure 4e) and electron energy loss spectroscopy (EELS) (Figure 5). Two locations on the lamella consisted of W_18_O_49_ and WO_3_ phases were probed in the direction of the nanowhisker growth along the ⟨010⟩ axis. Indeed, the W_18_O_49_ lattice displayed in Figure 4e and discussed below is appreciably more complex than the WO_3_ monoclinic lattice. Interestingly, the arrays of octahedra forming both WO_3_ and W_18_O_49_ phases are aligned along the same axis ⟨101⟩ ∥ ⟨103⟩, respectively. This is direct indication that the oxidized WO_3_ phase is grown alongside with the W_18_O_49_ phase and not oxidized afterward. This deduction is in close match with the *in situ* growth in TEM as shown in Figure 3 and the corresponding Video S5. Therefore, during the growth, multiple phases (W_18_O_49_; WO_3_ and other suboxides) are formed. From the regular view, which is perpendicular to the (010) plane, the distinction of such phases is challenging. However, some indications are present, visible as shear plane discrepancies (observed in Figure 3b). In conclusion, the grown W_18_O_49_ nanowhiskers were primarily composed of W_18_O_49_ lattice domains interspersed with tungsten suboxide phases possessing a higher oxygen content. The notable presence of structural defects, corresponding with higher tungsten suboxides, could potentially be attributed to the reaction conditions. While the low-pressure hydrogen atmosphere is favorable for the vaporization of the oxide, it may be insufficient for the complete reduction of W^VI^ to suboxides.

The oxygen-K edge of the energy loss near-edge structure (ELNES) spectrum of the pure W_18_O_49_ phase was acquired and compared with DFT calculations (Figure 5a). To collect the ELNES data for the W_18_O_49_ lattice, the focused beam was scanned through the entire area marked by the lower yellow square on the cross section (Figure 4e). This procedure ensured that the measurement was taken from a domain consisting entirely of the W_18_O_49_ phase. Other suboxide phases in the material could alter the spectrum, especially when viewed perpendicularly to the ⟨010⟩ axis. The complexity of the W_18_O_49_ lattice could be described in terms of the different types of tungsten and oxygen atoms in the structure. Specifically, each of the oxygen lattice sites and its coordination contribute differently to the oxygen-K edge ELNES. The W_18_O_49_ lattice is monoclinic (point group *P*2/*m*). The tungsten atoms have two oxidation states in this lattice (W^6+^ and W^5+^) and three coordination types (Figure 5b). The most prevalent one is octahedral [W^6+^O_6_] (black). Two tungsten atoms in oxidation state (6+) are in pentagonal bipyramidal conformation (brown). The third type of tungsten is in oxidation state (5+) and forms a W^5+^–W^5+^ pair (marked in Figure 5b as gray atoms and in Figure 5c encircled by a yellow oval line). From a symmetry point of view, there are multiple types of oxygen atoms. Roughly speaking, the oxygen atoms could be sorted into six groups to distinguish their contribution to the ELNES. The most abundant oxygen atoms are those bonded to tungsten (6+) (blue). The second most abundant oxygen atoms (orange) are “triple” bonded to tungsten (6+) (two of them are in an octahedral arrangement and one in a pentagonal bipyramidal arrangement). The next oxygen atoms (yellow) are “triple” linked to octahedral tungsten (6+), another octahedral tungsten (5+), and tungsten (6+) in a pentagonal bipyramid arrangement. The red oxygen atoms are bonded to two tungsten (5+) atoms and one (6+). Finally, the green and cyan oxygens are bonded to two tungsten atoms in the (010) direction: the green atom is bonded to tungsten (6+) in a pentagonal bipyramid and the cyan atom to two tungsten atoms (5+). In fact, one should distinguish between two groups of oxygen atoms. This grouping is evident in the DFT calculation of the ELNES spectrum of the oxygen K edge (Figure 5d). The blue, yellow, and orange oxygen atoms could be further divided into subgroups. However, to minimize the complexity of the presentation, they are represented each by one color. Nonetheless, their heterogeneity (second nearest neighbors) is manifested by the broadness of their calculated ELNES band-like spectrum (Figure 5d). In sharp contrast, the green, cyan, and red curves are narrow, reflecting the minor heterogeneity of the oxygen atoms bound to W 5+ atoms.

The DFT-calculated contribution of each kind of oxygen atom to the ELNELS spectrum is shown in Figure 5d. Indeed, the ELNES spectrum of the oxygen coordinated to W^5+^ and to W^6+^ in the pentagonal bipyramid (marked red, orange, and yellow) are substantially different from the rest of the oxygens, especially with regard to the local maxima around 4–8 eV together with relatively lower intensities of the peaks in 0–4 eV. The EELS spectrum was described by five line markings (Figure 5a). The most prominent signal (A) is formed predominantly by the octahedra of the O_w6+_ octahedra (blue), which are prevalent in the lattice. The signal (B) could be described as a shoulder in the 4–6 eV range and is considerably weaker since the oxygen atoms forming it are not so abundant (oxygens in pentagonal bipyramidal features and in coordination with a W^5+^–W^5+^ pair, orange, yellow, and red). The peak (C) consists of the contribution of oxygen atoms in [WO_6_] octahedra (blue), similar to the peak (A). The last attributed peak (D) consists of multiple oxygens but again mainly from the ones in octahedra (blue). The spectrum is therefore mainly formed from oxygen atoms in octahedral coordination. Yet, the specific acquisition from (010) or *b*-axis view could influence the ELNES spectrum due to diffraction^52^ effects and channeling.^53^ Therefore, the specific orientation of the acquisition may affect the ELNES fine structure. Such effects could explain the minor discrepancies between the calculated ELNES spectrum and the experimentally acquired spectrum (Figure 5a).

### 3.5 Preparation of W_18_O_49_/*a*-SiO_2_ Nanofibers – Multigram Synthesis in a Tube Furnace

**Figure 6:**
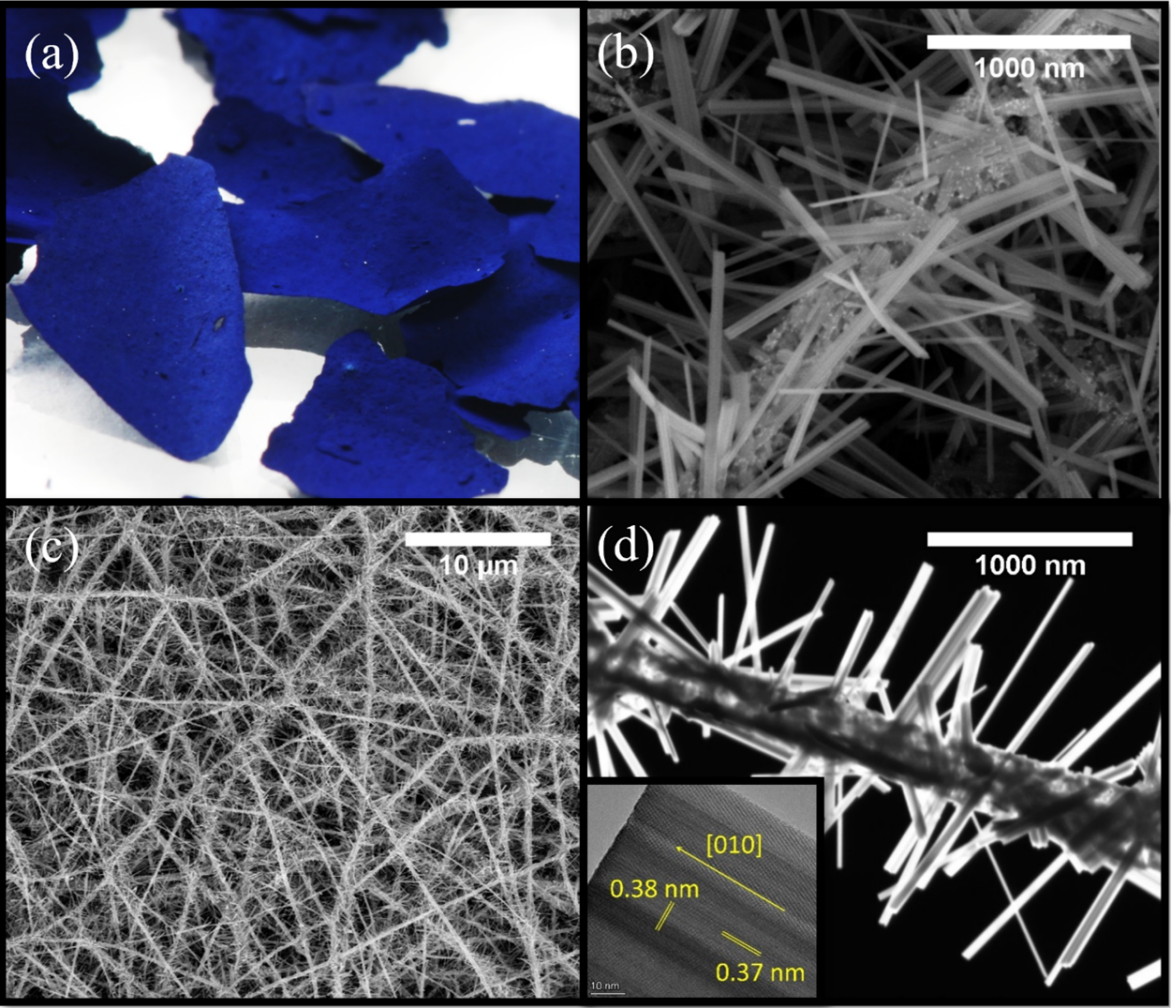
(a) Optical photograph, (b, c) SEM images in two magnifications, (d) STEM DF image of the W_18_O_49_/*a*-SiO_2_ nanofibers. The mean diameter of the nanofibers was 248 ± 72 nm. The mean length and diameter of the W_18_O_49_ nanowhiskers were 894 ± 363 and 45 ± 25 nm, respectively. The HRTEM image of the prepared nanowhisker shows interplanar distances corresponding to W_18_O_49_ (inset of d).^32^

Based on the observations and mechanistic studies carried out in the *in situ* SEM/TEM investigations, optimized growth conditions for the W_18_O_49_ nanowhiskers have been acquired. These parameters were further exploited in the multigram synthesis of nanowhiskers in a flow reactor. In particular, the reaction conditions of *in situ* SEM experiment No. 1 in the μReactor, i.e., 100 Pa of H_2_ and 800 °C, were utilized in a quartz tube reactor. The shock heat treatment performed in the quartz tube reactor with a preheated translating furnace (Figure S7) was used for the maximal achievable heating rate comparable to the one used in the MEMS chips in the μReactor. The yellowish flakes of the γ-WO_3_/*a*-SiO_2_ precursor nanofibers turned deep blue after the reaction in the flow reactor as a result of reduction by the heated hydrogen gas (Figure 6a). SEM analysis revealed an open nanofibrous structure, as shown in Figure 6b. Individual nanofibers formed as interconnected nonwoven web typical for electrospun materials. A detailed SEM observation, in Figure 6c, shows structures similar to those already observed in the μReactor during the *in situ* SEM experiments (compare with Figure 2). The W_18_O_49_ nanowhiskers grew from the nanofiber’s surface, forming urchin-like structures (described in the following text as W_18_O_49_/*a*-SiO_2_). The mean diameter of the nanofibers was 248 ± 72 nm, not including the length of the grown W_18_O_49_ nanowhiskers. Comparably, the mean diameter of the precursor γ-WO_3_/*a*-SiO_2_ nanofibers (Figure S5) was 225 ± 88 nm. The average length and thickness of the W_18_O_49_ nanowhiskers were 894 ± 363 and 45 ± 25 nm, respectively.

Scanning transmission electron microscopy in the dark field mode (STEM-DF) was used for chemical difference imaging (Z-contrast) of the nanowhiskers and the nanofibers (Figure 6d). The heavier atoms (W) of the W_18_O_49_ nanowhiskers in the STEM-DF appear brighter than the silica-based nanofibers with lower atomic numbers (Si). Therefore, the concentration of tungsten is higher in the grown nanowhiskers compared to that in the nanofibers. Additionally, W_18_O_49_/*a*-SiO_2_ nanofibers prepared in the flow reactor were analyzed by HRTEM. The distances observed in the HRTEM between the two pairs of perpendicular crystal planes were 3.8 and 3.7 Å, as shown in the insert in Figure 6d. These distances correspond with the spacing of crystal planes perpendicular and parallel to the (010) crystal plane of W_18_O_49_ nanowhiskers, respectively.^32^

From a chemical perspective, the precursor nanofibers consist of γ-WO_3_ and *a*-SiO_2_ phases. The W_18_O_49_ nanowhiskers grew from the γ-WO_3_/*a*-SiO_2_ nanofibers via the transport of volatile tungsten species (conceivably WO_2_(OH)_2_).^3^ Previously, the high-temperature reduction of γ-WO_3_/*a*-SiO_2_ nanofibers at atmospheric pressure resulted in metallic tungsten nanoparticles interconnected by amorphous silica to the nanofibrous structure.^41^ In the present study, we observed the W_18_O_49_ nanowhisker’s growth under the low-pressure hydrogen atmosphere, while the amorphous silica remained chemically intact in the nanofiber. The XRD pattern (see Supporting Information Discussion 4, Figure S14) of the W_18_O_49_/*a*-SiO_2_ nanofibers corresponded mostly to the diffractions of substoichiometric tungsten oxide W_18_O_49_ (ICSD-202488). The Rietveld refinement analysis of the diffractogram also identified a small portion (12%) of tungsten suboxide W_50_O_148_ (ICSD-77709) which could be associated with oxidized areas revealed by cross-sectional analyses in Figures 5 and 6. STEM-HAADF and STEM-EDS were utilized for the chemical and structural analysis of the nanofiber/nanowhisker interface (Supporting Information Discussion 4, Figure S15). Additional experiments in the tube furnace under vacuum were performed for comparison with the μReactor within SEM (see Figure S16) showing a close match of both approaches.

## 4 Conclusions

Herein, the growth of W_18_O_49_ nanowhiskers from γ-WO_3_/*a*-SiO_2_ nanofibers was studied in-depth. Three main approaches were utilized to obtain a fundamental understanding of the reaction:

•The new μReactor technology allowed *in situ* SEM observation of the nanowhisker growth from γ-WO_3_/*a*-SiO_2_ nanofibers at elevated temperatures and under hydrogen atmosphere. Additionally, the formation of nanowhisker bundles was described as a time-resolved process *in situ*. The e-beam was found to influence the nanowhiskers’ growth. In particular, nanowhiskers that were exposed to the e-beam were found to grow slower than the nonirradiated ones. Thus, the *in situ* growth in the SEM technique provided valuable insights on the effective reaction conditions (temperature, hydrogen pressure, and reaction time), landing itself into a highly effective analytical and development tool.

•*In-situ* TEM experiments at elevated temperatures followed in detail the structural changes during the W_18_O_49_ nanowhisker growth at elevated temperatures and under deep vacuum conditions. Notably, shear planes formation and translation along the ⟨ 010⟩ axis could be followed directly. HRSTEM analysis of a cross-section of W_18_O_49_ nanowhiskers was carried out. An ordered superstructure of pentagonal columns and hexagonal channels typical of the Magnéli phases was clearly revealed. Additionally, oxidized W-O phases were found next to the W_18_O_49_ lattice. The crystalline structure of the observed phases was investigated by nanodiffraction. Moreover, ELNES spectrum on O-K edge was acquired from the pure W_18_O_49_ phase. Strong diffraction and channeling effects were found to possibly influence the spectrum due to observation strictly parallel with (010) plane. DFT calculated spectrum and individual contributions of oxygens to the ELNES signal of the O-K edge were acquired. These analyses paves the way for the future microscopical investigation of the tungsten suboxides’ fundamental characterizations.

•Finally, knowledge gained from the *in situ* SEM and TEM experiments was used for the development of a multigram synthesis of W_18_O_49_/*a*-SiO_2_ nanofibers with a unique urchin-like structure. The upscaled reaction was performed in a tube furnace under comparable conditions to those used in the SEM-fitted μReactor (800 °C, 100 Pa of H_2_). The successful synthesis of the nanowhiskers in the flow system proved the effectiveness of the complementary *in situ* SEM/TEM studies (both using the MEMS chip technology) and the traditional tube furnace processing as an accelerated reaction development and optimization method.

## Supporting Information Available

Schematics of the electrospinning apparatus, Preparation of γ-WO_3_/a-SiO_2_ nanofibers, Density Functional Theory Calculations, *In-situ* Reaction in the TEM Using the MEMS Chip, Heat Treatment – Partial Reduction in a Tube Furnace, Kinetic Study of Growth of W_18_O_49_ Nanowhiskers *in situ* in the SEM, Partial Reduction of γ-WO_3/a_-SiO_2_ Nanofibers at 900 °C under Vacuum (1.10^–4^ Pa), *In-situ* Reaction in the TEM Using the MEMS Chip, Preparation of W_18_O_49_/SiO_2_ Nanofibers – Multigram Synthesis in a Tube Furnace, STEM-HAADF/EDS Analysis of W_18_O_49_/α-SiO_2_ Nanofiber (PDF)
e-Beam of the SEM focused on an individual nanofiber (AVI)
Partial reduction of γ-WO_3_/α-SiO_2_ nanofibers at 900 °C under vacuum) (AVI)
Growth in the *in situ* TEM experiment acquired in real-time (AVI)
Shear planes progressing along both the ⟨010⟩ and the opposite ⟨0–10⟩ directions (AVI)
Cropped from the full size Video S7 for clarity (AVI)
Reactive growth continuation of Video S7 (AVI)
Shift of the shear plane observed *in situ* in TEM on a W_18_O_49_ nanowhisker (AVI)

## Supplementary Material

cg3c01094_si_001.pdf

cg3c01094_si_002.avi

cg3c01094_si_003.avi

cg3c01094_si_004.avi

cg3c01094_si_005.avi

cg3c01094_si_006.avi

cg3c01094_si_007.avi

cg3c01094_si_008.avi
