# Supplementary material for: W18O49 Nanowhiskers Decorating SiO2 Nanofibers: Lessons from In Situ SEM/TEM Growth to Large Scale Synthesis and Fundamental Structural Understanding
Source: Cryst Growth Des. 2023 Dec 5;24(1):378–90. doi: 10.1021/acs.cgd.3c01094 (PMC10767701; doi:10.1021/acs.cgd.3c01094)
Supplement: Supplementary file 1 — cg3c01094_si_001.pdf [file cg3c01094_si_001.pdf]

Supplementary Information

$W_{18}O_{49}$ Nanowhiskers Decorating SiO_2 Nanofibers: Lessons from *in-situ* SEM/TEM Growth to Large Scale Synthesis and Fundamental Structural Understanding

Vojtech Kundrat[a,b,c] Kristyna Bukvisova[b,d] Libor Novak[b] Lukas Prucha[e] Lothar Houben[a] Jakub Zalesak,[b,f] Antonio Vukusic[g] David Holec[g] Reshef Tenne,[a] and Jiri Pinkas*,[c]*

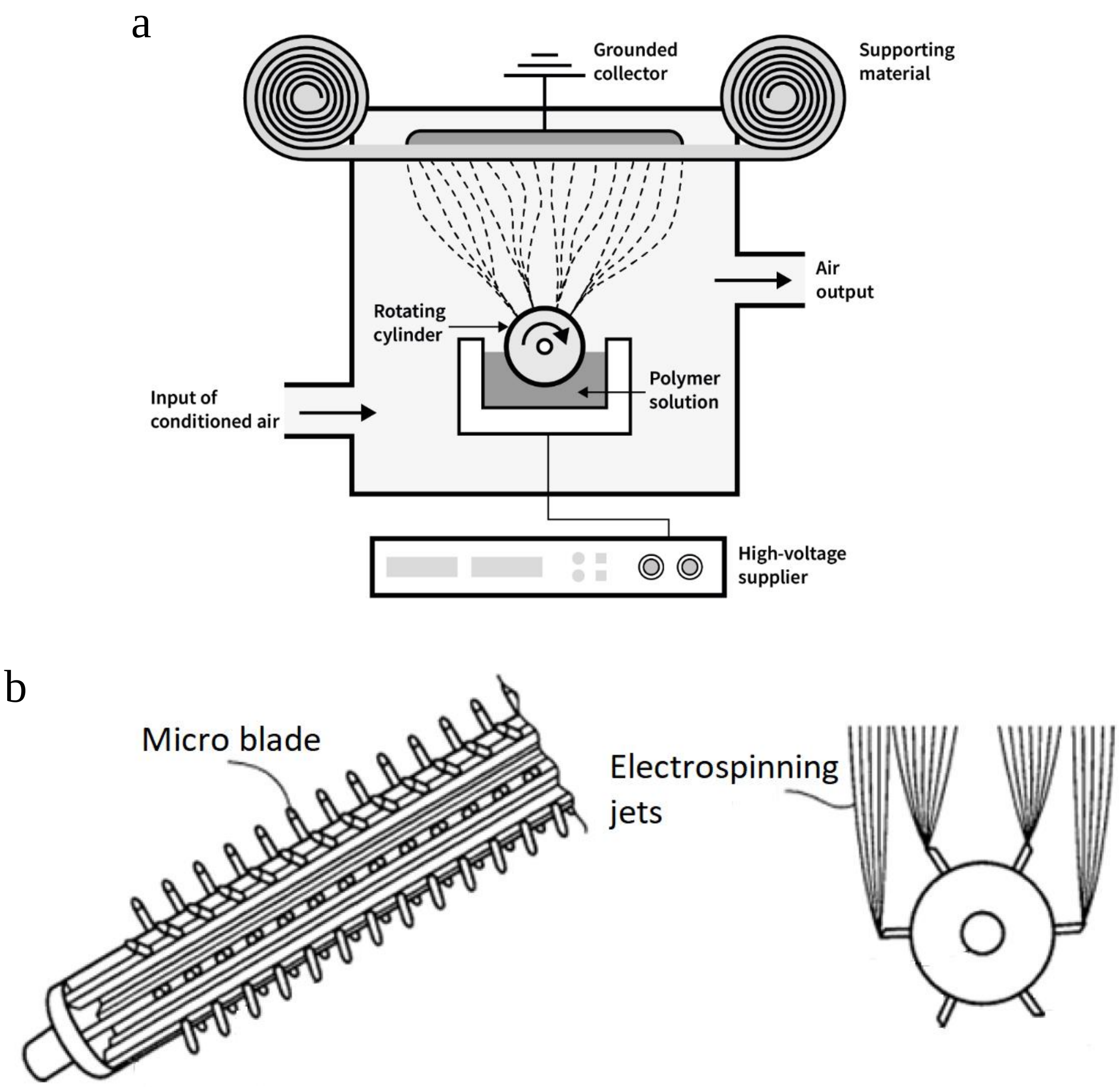

Figure S1. a) Scheme of Nanospider NS LAB500S (Elmarco, Czech Republic) electrospinning setup used for production of PVA-silicotungstic acid nanofibers. b) The cylindrical electrode compartment with micro blades for allocation of electrical charge and solution droplets.[42]

Supplementary Experimental Part 1

Preparation of γ-WO_3/a-SiO_2 Nanofibers

PVA (150 g) was dissolved in deionized water (1150 g) by stirring and heating for several hours providing an 11.5 wt% solution. Silicotungstic acid hydrate (120 g) was dissolved in deionized water (200 g). Both solutions were combined at ambient temperature and homogenized by intensive stirring for several hours. The final polymer and tungsten precursor contents in the prepared solution were approx. 9.3 and 7.4 wt%, respectively.

Thermogravimetric analysis and differential scanning calorimetry (TG/DSC) were performed on a Netzsch Jupiter STA 449 instrument with a heating rate of 10 K min^{-1} and a maximum temperature of 1000 °C.

The prepared solution (approx. 800 cm^3) was transferred to the electrode vessel with a partially submerged electrode. A grounded counter electrode in the form of a stretched wire was covered with a large sheet of aluminum foil (approx. 0.8 x 0.4 m), and the electrode distance was set to 17.0 cm. The speed of the rotating electrode was set to 30 rpm. The applied voltage was set to 50 kV. The process was running for 4 h until one half of the solution was consumed, and the aluminum collector was covered by a thick layer of nonwoven felt of green composite of PVA and silicotungstic acid. The prepared fibrous material was peeled off, analyzed by SEM (**Figure S2**) and TGA/DSC (**Figure S3**) and used in further process.

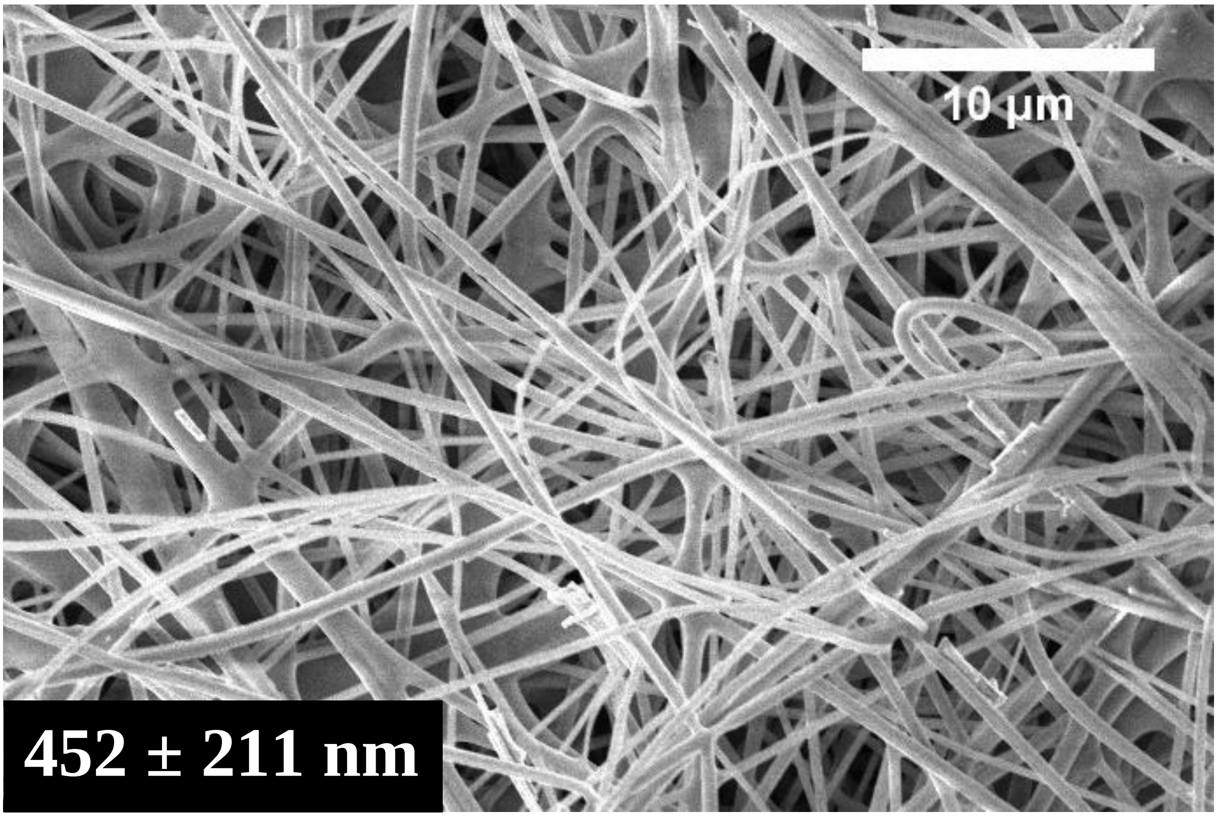

Figure S2. SEM image of green nanofibrous composite of PVA and silicotungstic acid.

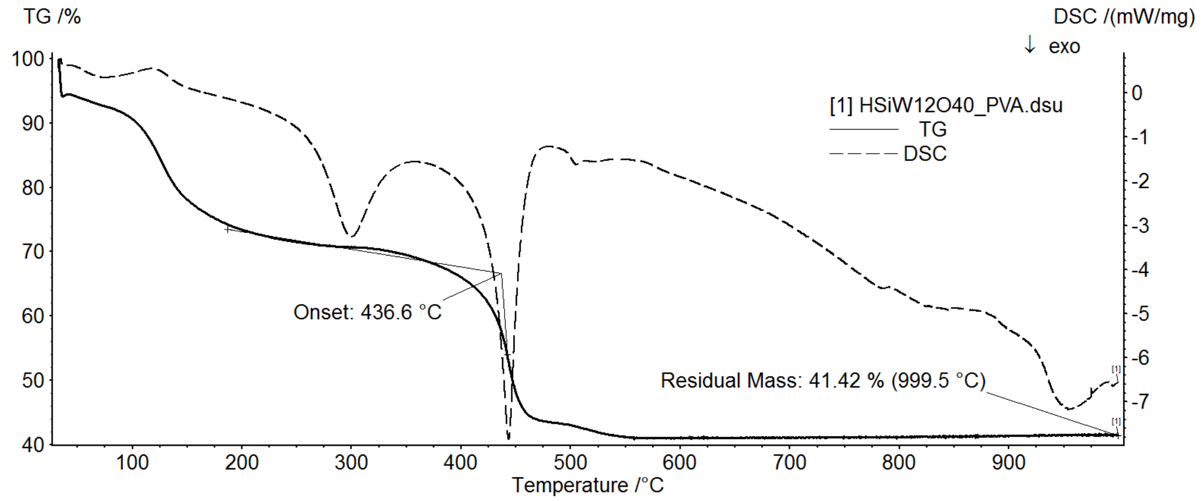

Figure S3. TG/DSC traces of green composite of PVA and silicotungstic acid measured in air.

The green fibers from PVA and HSiW were calcined in air at 600 °C in a muffle furnace. The furnace was heated during 1 h to the final temperature followed by 2 h of dwell time. After heat treatment, the sample was left to cool down spontaneously to ambient temperature. The nanofibrous γ-WO_3/*a*-SiO_2 material was analyzed by X-ray diffraction (XRD) (**Figure S4**) and SEM (**Figure S5**).

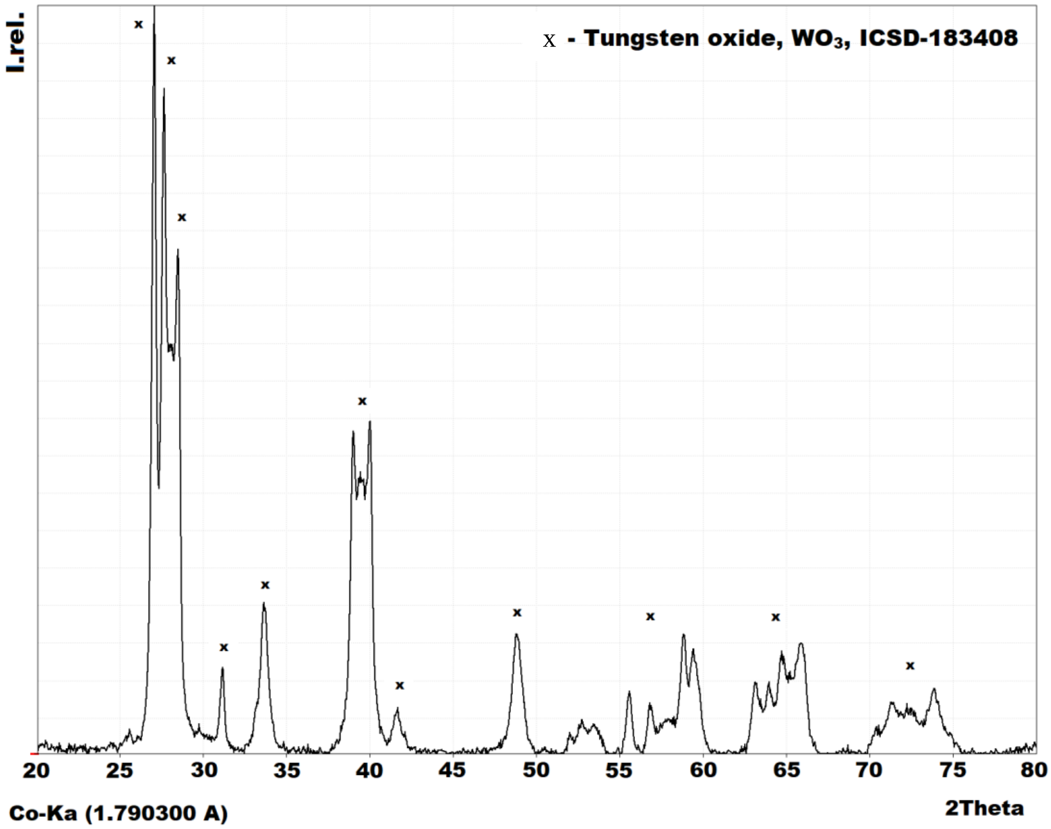

Figure S4. X-ray powder diffractogram of the γ-WO_3/*a*-SiO_2 precursor nanofibers. The peaks of the WO_3 phase are marked by x. All the peaks of the diffractogram belong to a single WO_3 phase (low intensity or signal in close proximity to large peaks were not marked for clarity).

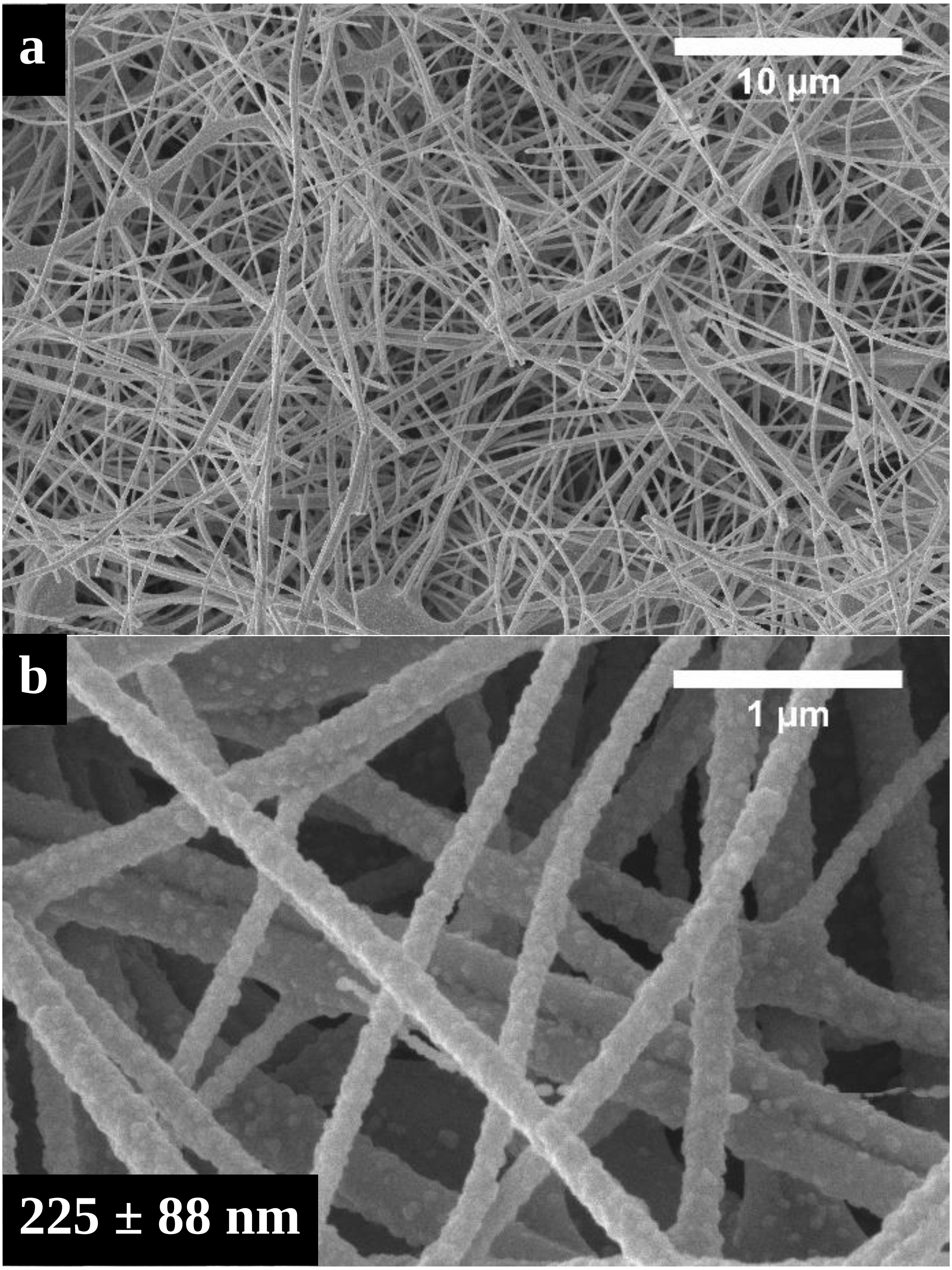

Figure S5. a) SEM image of γ-WO_3/*a*-SiO_2 nanofibrous materials calcined at 600 °C and b) its magnified view.

Supplementary Experimental Part 2

Density Functional Theory Calculations

To model $W_{18}O_{49}$, we took the structural model reported in the Materials Project [1], entry mp-907, from database version v2022.10.28. To calculate ELNES, we used density functional theory [2,3] as implemented in the all-electron Wien2k code [4]. The exchange and correlation interactions were treated at the GGA-PBE level [5]. Spherical harmonics basis up to $l = 10$ was employed for expanding the wave functions inside the non-overlapping muffin-tin (MT) spheres, whereas the plane-wave cut-off energy was defined by $R_{MT}.k_{max} = 7$. The MT radii were automatically set by structGen (a part of the Wien2k package) to values of 1.84 a.u. and 1.59 a.u. for W and O, respectively. We used a dense mesh of 12x15x53 *k*-points to discretize the whole first Brillouin zone of the unit cell. We used the Telnes package (a part of the Wien2k) to calculate the O K-edge ELNES. We calculated the ELNES on every symmetry-inequivalent O atom, and the resulting curves were averaged using the atom multiplicities, a method previously applied for systems with many local environments [6]. We applied an artificial broadening of 0.5 eV to the calculated raw ELNES spectra to simulate experimental spectra.

[1] A. Jain, S.P. Ong, G. Hautier, W. Chen, W.D. Richards, S. Dacek, S. Cholia, D. Gunter, D. Skinner, G. Ceder, K.A. Persson, Commentary: The Materials Project: A materials genome approach to accelerating materials innovation, APL Materials. 1 (2013) 011002.

[2] P. Hohenberg, W. Kohn, Inhomogeneous electron gas, Physical Review. 136 (1964) B864–B871.

[3] W. Kohn, L.J. Sham, Self-consistent equations including exchange and correlation effects, Physical Review. 140 (1965) A1133–A1138.

[4] P. Blaha, K. Schwarz, F. Tran, R. Laskowski, G.K.H. Madsen, L.D. Marks, WIEN2k: An APW+lo program for calculating the properties of solids, J. Chem. Phys. 152 (2020) 074101.

[5] J.P. Perdew, K. Burke, M. Ernzerhof, Generalized Gradient Approximation Made Simple, Phys. Rev. Lett. 77 (1996) 3865–3868.

[6] D. Holec, R. Rachbauer, D. Kiener, P.D. Cherns, PMFJ Costa, C. McAleese, P.H. Mayrhofer, C.J. Humphreys, Towards predictive modeling of near-edge structures in electron energy-loss spectra of AlN-based ternary alloys, Phys. Rev. B Condens. Matter. 83 (2011) 165122.

In-situ Reaction in the TEM Using the MEMS Chip

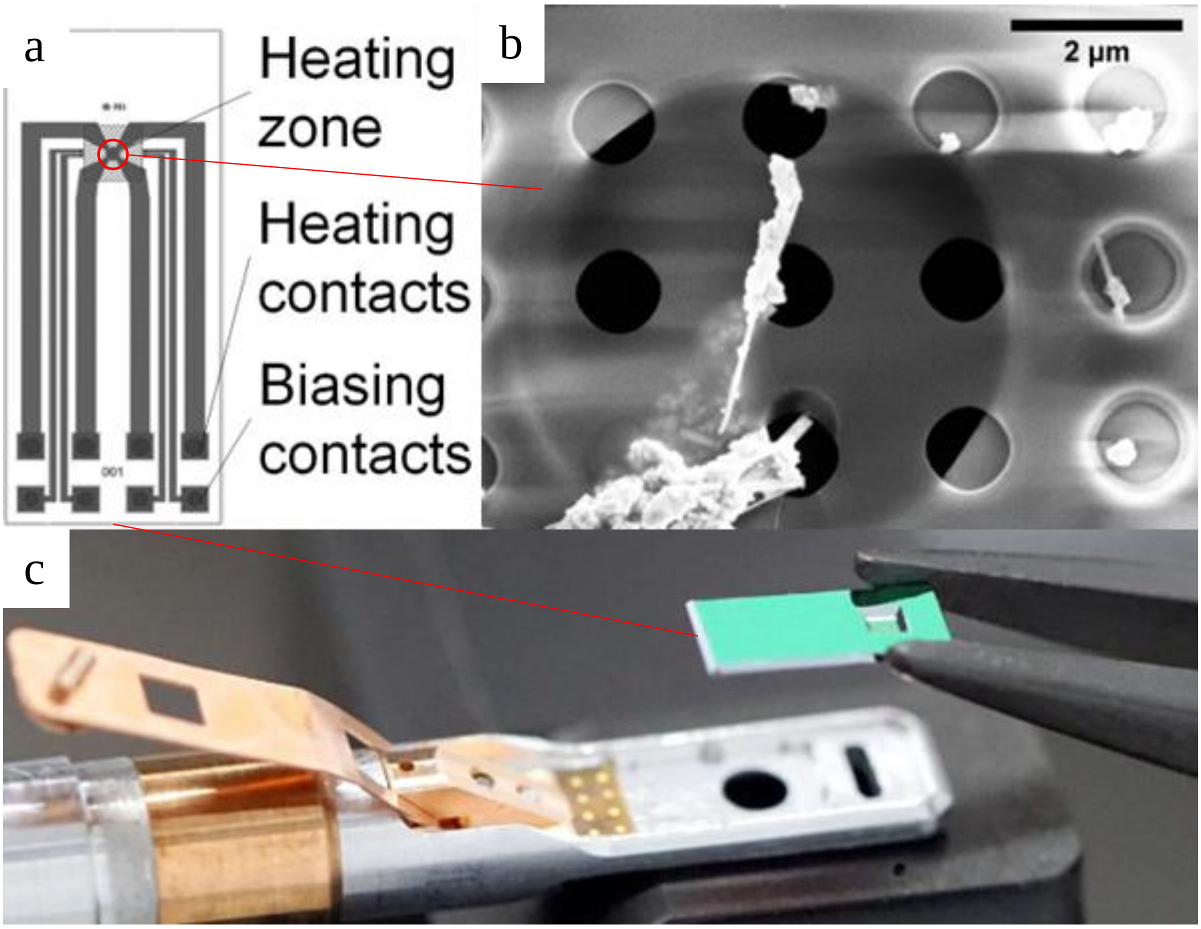

Figure S6. a) Scheme of the MEMS chip used for the *in-situ* TEM experiments. b) SiC membrane on the MEMS heating chip. c) Thermo Fisher Scientific NanoEx-i/v heating and biasing holder for *in-situ* TEM. The heating zone consists of multiple circular areas fabricated on the SiC membrane (b) allowing *in-situ* TEM measurements. The sample was deposited on the membrane by dip coating of the chip in a suspension of the studied material.

Heat Treatment – Partial Reduction in a Tube Furnace

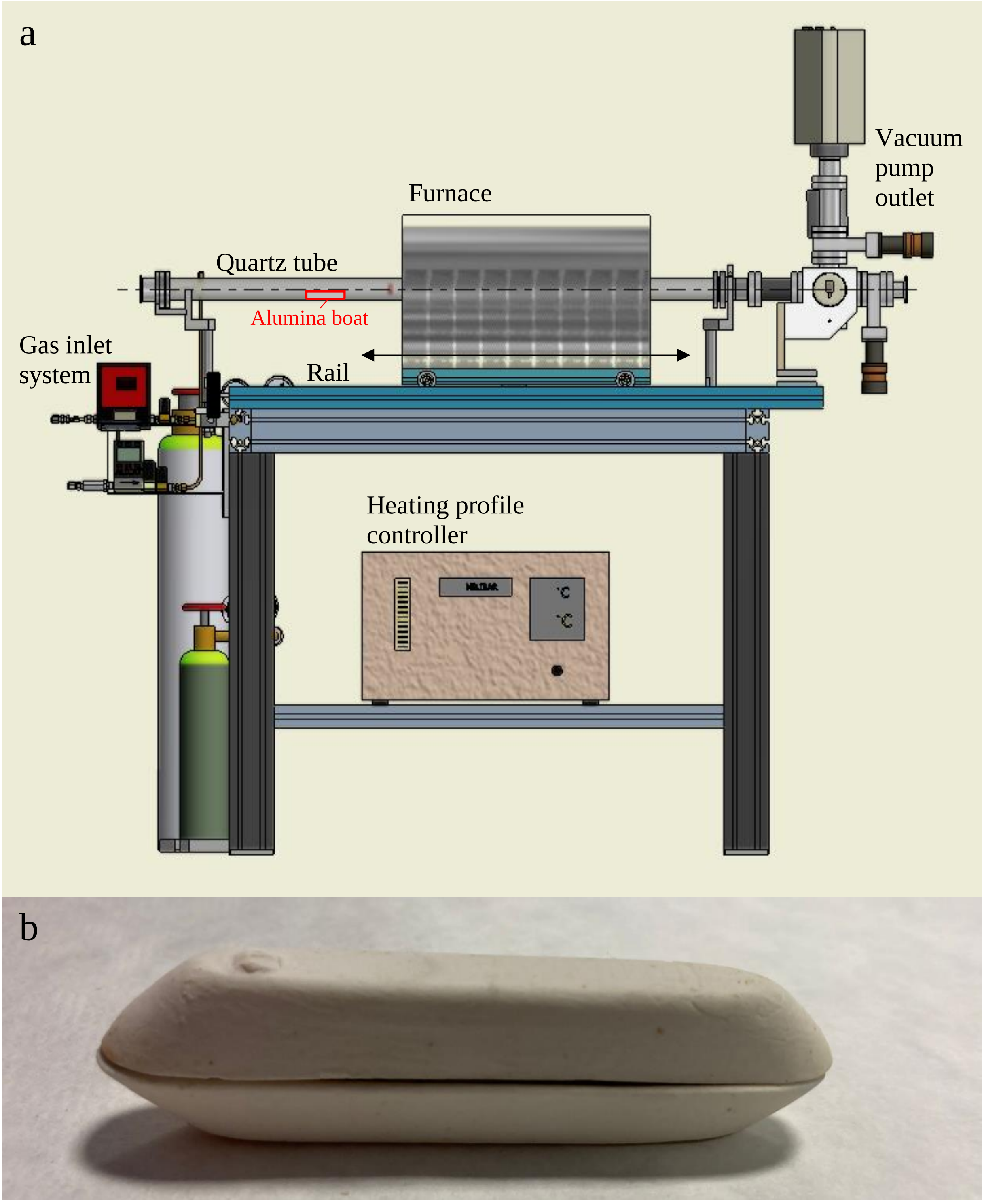

Figure S7. a) Custom-made tube furnace allowing shock heat treatment. b) Picture of the alumina boat used for placing the samples covered by another alumina boat. The setup furnace has a gas inlet and a vacuum pump as an outlet, enabling precise gas pressure control and vacuum annealing. The furnace is fixed on a rail allowing its translation, providing thus means for shock heat treatment. The alumina boat with the γ-WO_3/*a*-SiO_2 precursor nanofibers was covered with another alumina boat to prevent spill during the evacuation procedure.

Supplementary Discussion 1

Kinetic Study of Growth of $W_{18}O_{49}$ Nanowhiskers in-situ in the SEM

Following **Video 1**, a kinetic study of the growth of $W_{18}O_{49}$ nanowhiskers on the surface of an e-beam irradiated γ-WO_3/a-SiO_2 nanofiber in the μReactor within SEM was undertaken **(Figure S8)**. Two nanowhiskers (marked A and B), which exhibited some differences in their growth mode are shown (at 800 °C and under 100 Pa of H_2 gas). Note that both nanowhiskers changed their lateral size (thickness) while growing in length in the μReactor. The discrepancy between the growth rates in the linear part and the final dimensions of the two $W_{18}O_{49}$ nanowhiskers is displayed in **Figure S8** and in **Table S1**, respectively.

Table S1. Comparison between the growth kinetics of two different e-beam irradiated $W_{18}O_{49}$ nanowhiskers

Nanowhisker	Maximal length [nm]	Maximal thickness [nm]	Growth rate [$nm.s^{-1}$]*
A	260	22	2.7
B	180	40	0.9

*- growth rate calculated from the linear fit of the length-time dependence

The length-time dependence of the growing nanowhiskers is shown in **Figure S8**. Both studied nanowhiskers grew initially at approximately a linear rate. The initial growth rate of the 22 nm nanowhisker (A) was three times faster than the 40 nm specimen (B). Bundles were formed by the emergence of neighboring nanowhiskers seeded by the primary nanowhisker.[43] However, the resulting morphology of the formed structures differed. The nanowhisker A appears inhomogeneous, with a bundle of nanowhiskers of different lengths grown in the same direction. While some grow with time, others disappear, likely due to Ostwald ripening. Alternatively, the diminishing of some nanowhiskers could be attributed to the combined effect of the heating and the high vacuum in the chamber, which could pump out the tungsten oxide vapors. The primary nanowhisker could be distinguished in the bundle as the longest (see arrow in **Figure S8 4A-8A**). On the contrary, bundle B was appreciably more compact and appeared as a single nanowhisker consisting of several domains with well-defined grain boundaries. The multidomain pattern of bundle B is more challenging to observe (in SEM), and only the thickening of the nanowhisker suggests its bundle-like character. Interestingly, both structures underwent shrinking at the last phase of the reaction. The original nanowhisker in bundle A

shortened quite significantly after 650 s. On the other hand, bundle B slimmed down and its length shortened only slightly at the end of the *in-situ* annealing process.

The *in-situ* growth process of the nanowhisker consists of two counter-acting fluxes. The heated γ-WO_3/*a*-SiO_2 provides a large local concentration of volatile WO_{3-x} clusters, which condense on the $W_{18}O_{49}$ tip and lead to its rapid growth. On the other hand, the strong vacuum pumping of the SEM and the heating effect of the e-beam lead to evaporation of WO_{3-x} clusters from the nanowhisker tip and its shortening. Initially (100-200 s), the latter effect is insignificant because the massive supply of WO_{3-x} clusters from the root of the γ-WO_3/*a*-SiO_2 nanofiber. However, as the nanowhisker gets longer, this supply diminishes and the pumping/e-beam heating of the nanowhisker leads to evaporation of WO_{3-x} clusters from its tip and shortening. In the meantime, new nanowhiskers start to grow nearby forming a bundle, due likely to this site being a "fertile ground" for such growth.

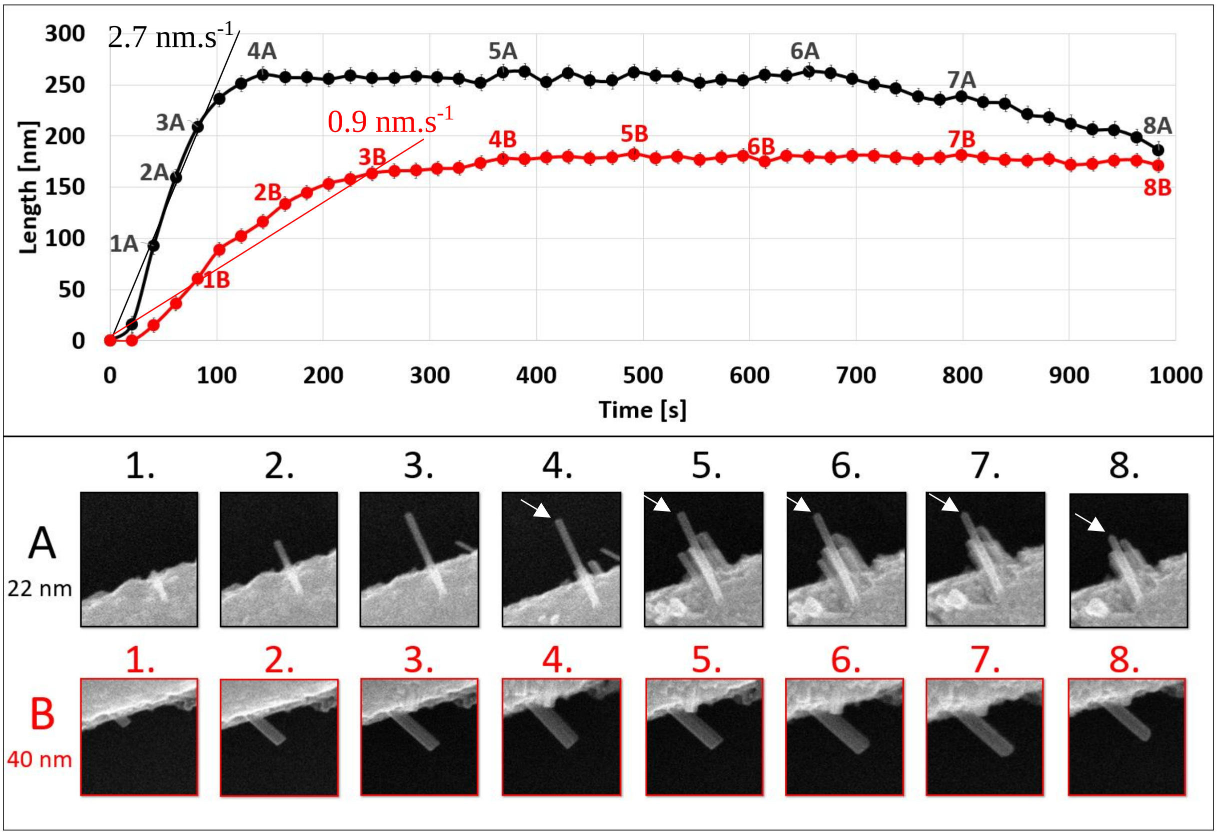

Figure S8. Kinetic analysis of growth of selected nanowhiskers. The 22 nm nanowhisker (A) was labeled 1-8A. Sequence 1-8B displays the growth mode of nanowhisker B. The fitting of the initial growth rate is (1-3A) 2.7 nm s^{-1}. Comparably, the initial growth rate of the nanowhisker B was only 0.9 nm s^{-1}.

Supplementary Discussion 2

Experiment No. 2 - Partial Reduction of γ-WO_3/a-SiO_2 Nanofibers at 900 °C under Vacuum (1.10^{-4} Pa)

In the subsequent experiment, the reaction was repeated in the SEM under vacuum and visualized in **Video 2** and **Figure S9a**. Compared with experiment No. 1, the nanowhiskers did not grow at 800 °C. Therefore, the temperature was elevated, and the nanowhisker growth started at 900 °C (**Figure S9a**, $t_{reaction}$ = 247 s, t_{video} = 6 s). The transformation was swift, producing short and bulky bundles (**Figure S9a**, t = 288-452 s, t_{video} = 6-10 s). The surface of the fiber did not undergo the reshaping into stacked bundles of nanowhiskers but instead became rough and coarsened. The irradiated bundles grew at a mean linear rate of 1.45 nm s^{-1}. At the same time, the fibers which were not irradiated by the e-beam took a different reaction path (**Figure S9b**). The resulting material appeared inhomogeneous at the end of the reaction. On the one hand, a part of the material had grown into long nanowhiskers and bundles. Contrarily, the rest of the observed sample in **Figure S9b** was a polycrystalline fibrous material (most likely a reduced tungsten whisker). The intense heating of the sample allowed accelerated nanowhisker growth; however, the process could not be adequately controlled.

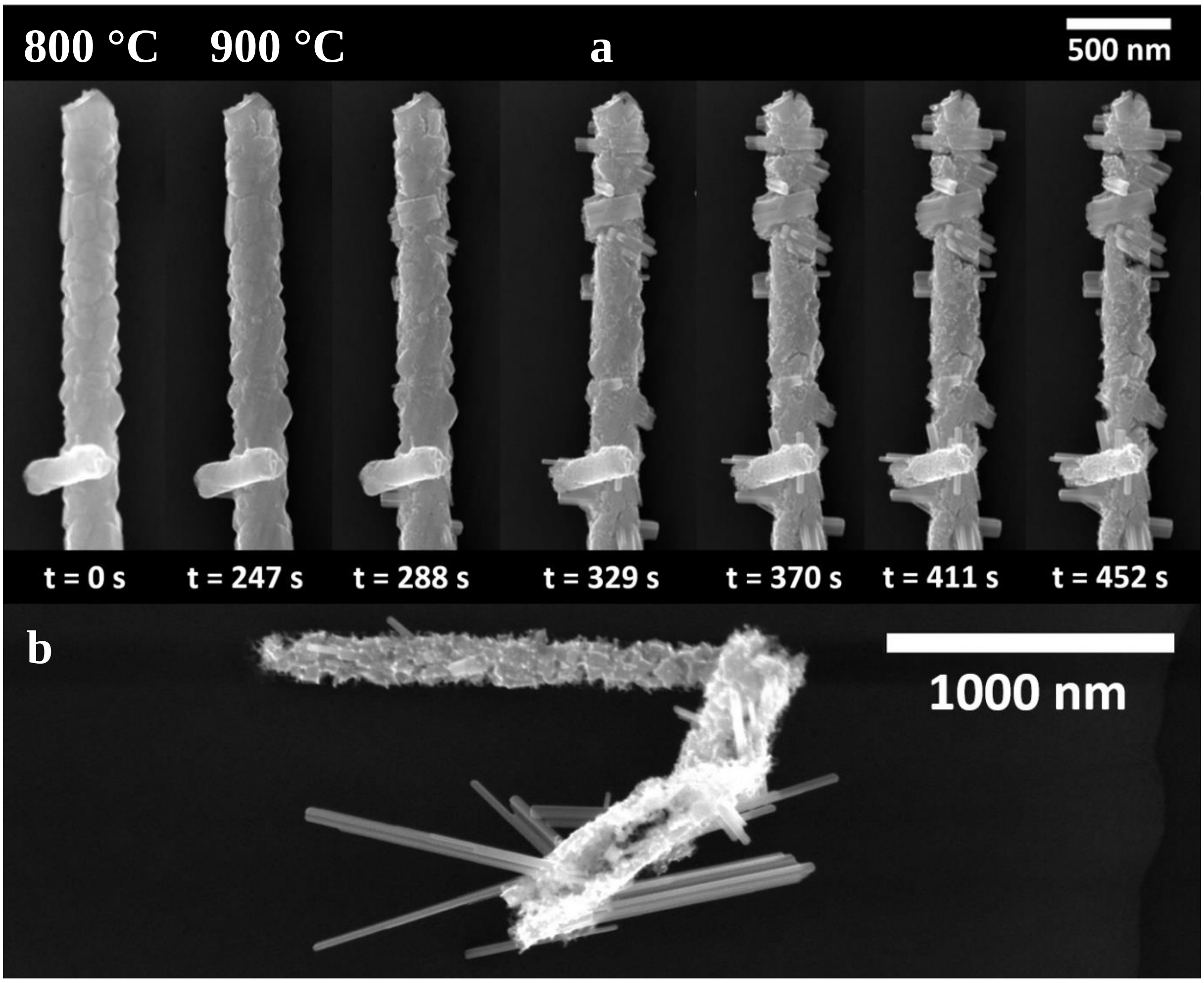

Figure S9. a) SEM images of the γ-WO_3/*a*-SiO_2 nanofibers heat treated in the μReactor within SEM (maximum temperature 900 °C, vacuum 1.10^{-4} Pa). b) Non-irradiated area by e-beam after the reaction. Individual images (a) were selected from **Video 2**. The growth and structural change started at 900 °C ($t_{reaction}$ = 247 s, t_{video} = 1-2 s). The fiber surface coarsened swiftly along with the formation of a crystalline phase ($t_{reaction}$ = 288 s, t_{video} = 3 s), which eventually transformed into distinctive short bundles ($t_{reaction}$ = 329-452 s, t_{video} = 4-8 s). The part of the specimen which was not irradiated, was analyzed after the reaction (b). Its structure was found to be inhomogenous with relatively long and thin nanowhiskers and crude bulky fibers.

Supplementary Discussion 3

In-situ Reaction in the TEM Using the MEMS Chip

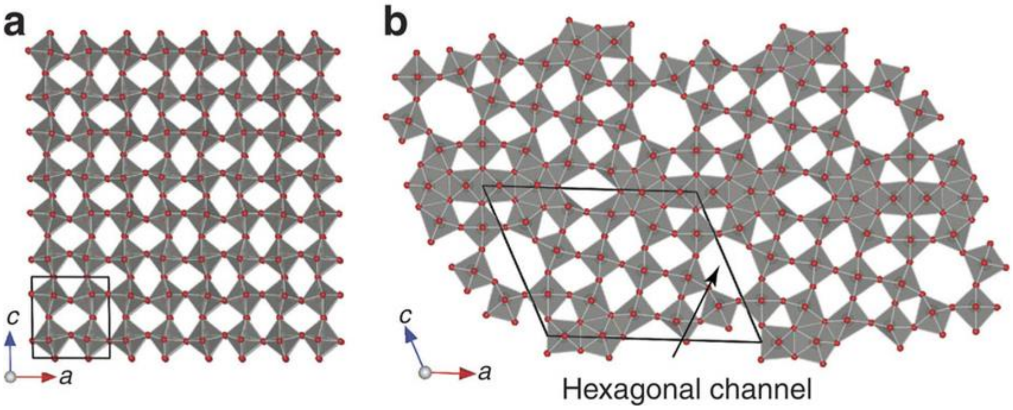

Figure S10. a) Structures of γ-WO_3 and b) $W_{18}O_{49}$. γ-WO_3 is formed by an array of corner-sharing [WO_6] octahedra with a monoclinic unit cell. $W_{18}O_{49}$ adopts a unique monoclinic structure of assembled pentagonal [WO_7] bipyramids and hexagonal channels constructed from six [WO_6] octahedra. Figure available via license CCBY4.0. Reprinted from S. Cong, Y. Yuan, Z. Chen, J. Hou, M. Yang, Y. Su, Y. Zhang, L. Li, Q. Li, F. Geng, Z. Zhao, *Nat. Commun.* **2015**, *6*, 7800.

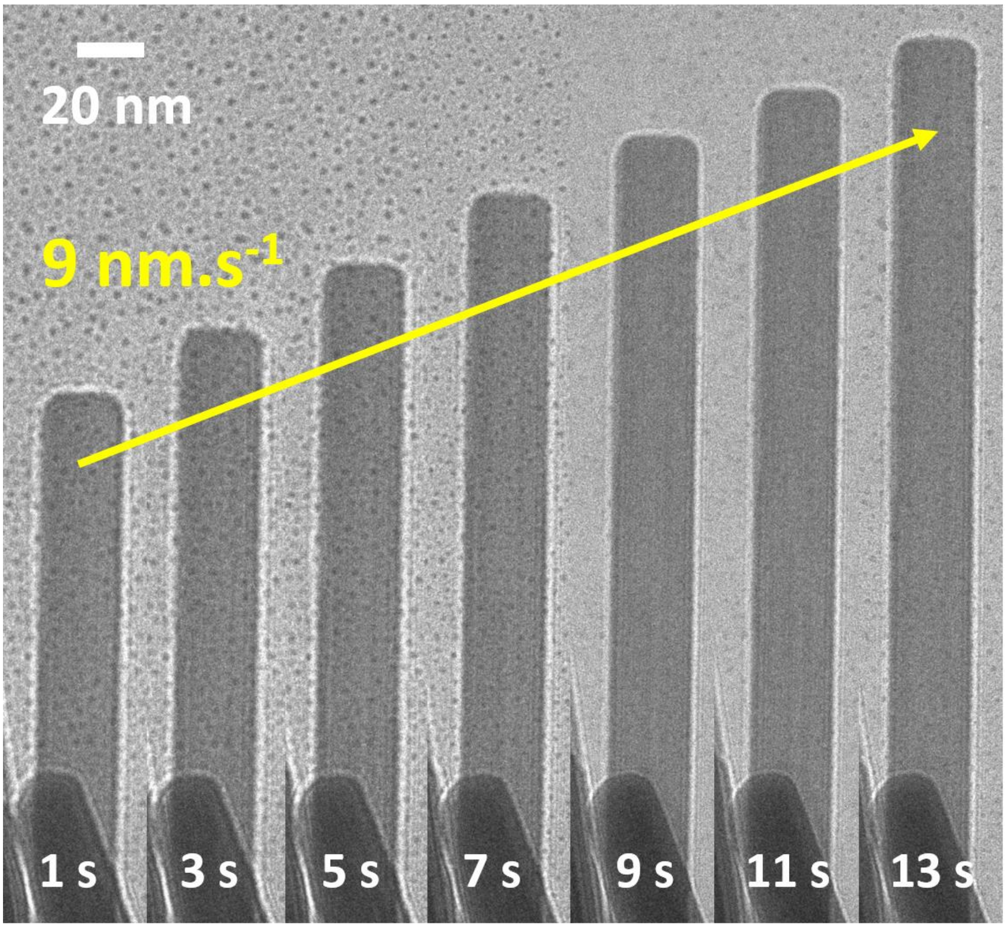

Figure S11. *In-situ* TEM analysis of the $W_{18}O_{49}$ nanowhisker growth at 820 °C (under e-beam irradiation). The growth rate was linear at 9 nm s^{-1}. The nanowhisker growth was observed out of focus in order to minimize the influence of the e-beam on the whisker growth.

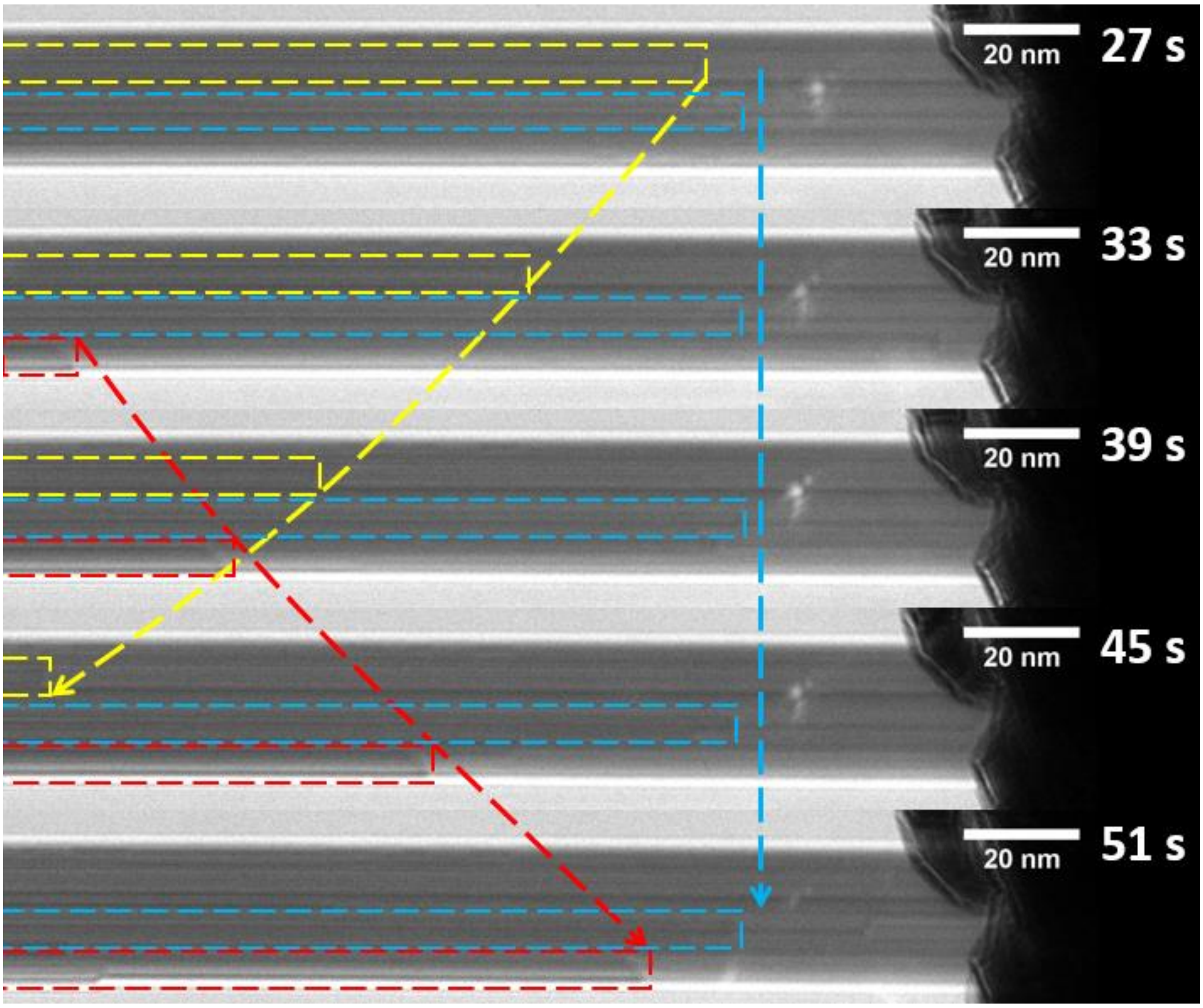

Figure S12. *In-situ* TEM images of shear plane translation in the structure of the growing $W_{18}O_{49}$ nanowhisker at 820 °C (under e-beam irradiation). Three shear planes were observed simultaneously, marked red, yellow, and blue. The red shear plane translates along the <010> crystal axis towards the nanowhisker base. On the other hand, the yellow-marked shear plane shifts in the opposite direction simultaneously. Finally, the blue-marked shear plane is stationary.

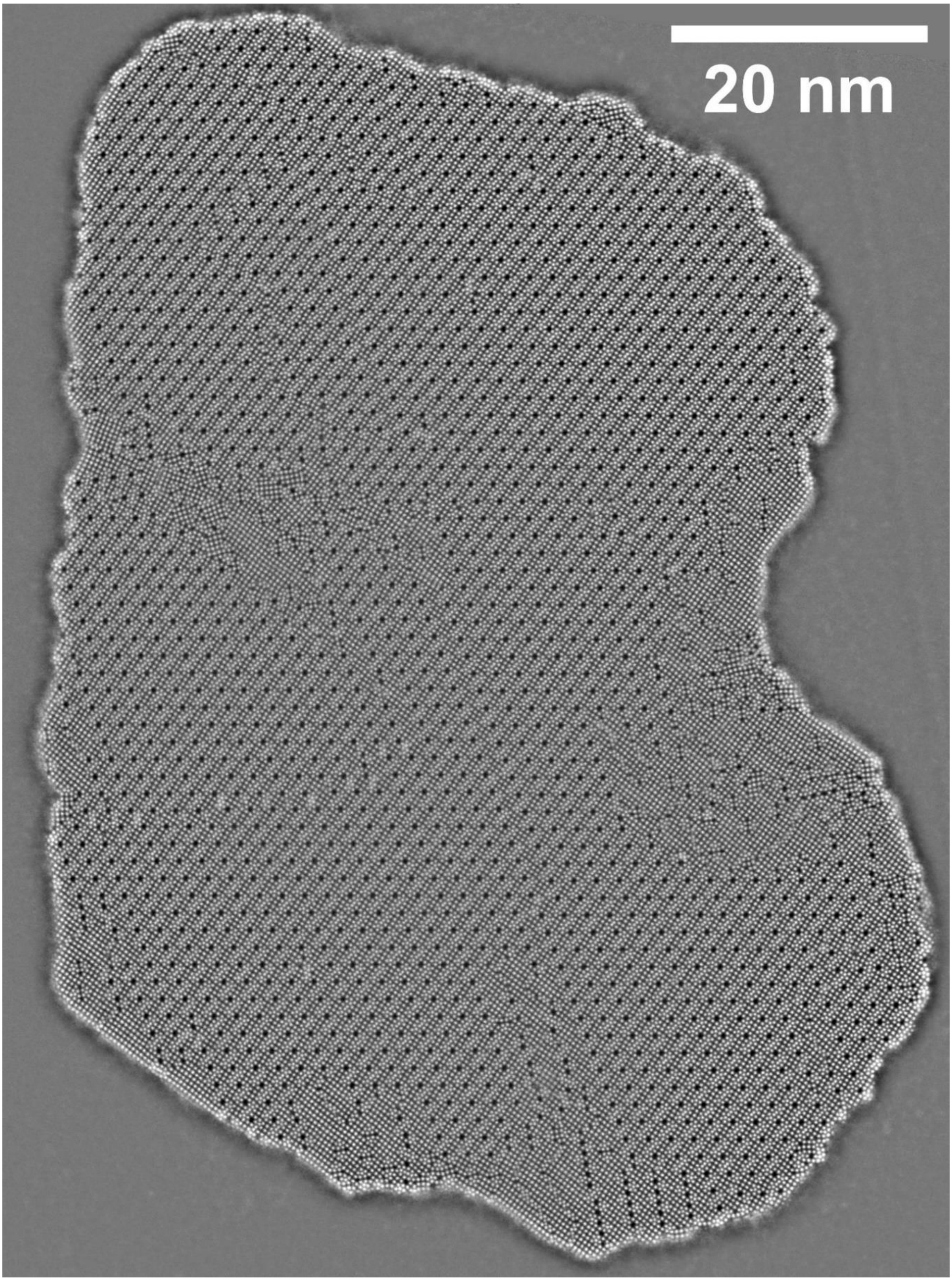

Figure S13. HRSTEM-HAADF analysis of the $W_{18}O_{49}$ nanowhisker cross-section.

Supplementary Discussion 4

Preparation of $W_{18}O_{49}/SiO_2$ Nanofibers – Multigram Synthesis in a Tube Furnace

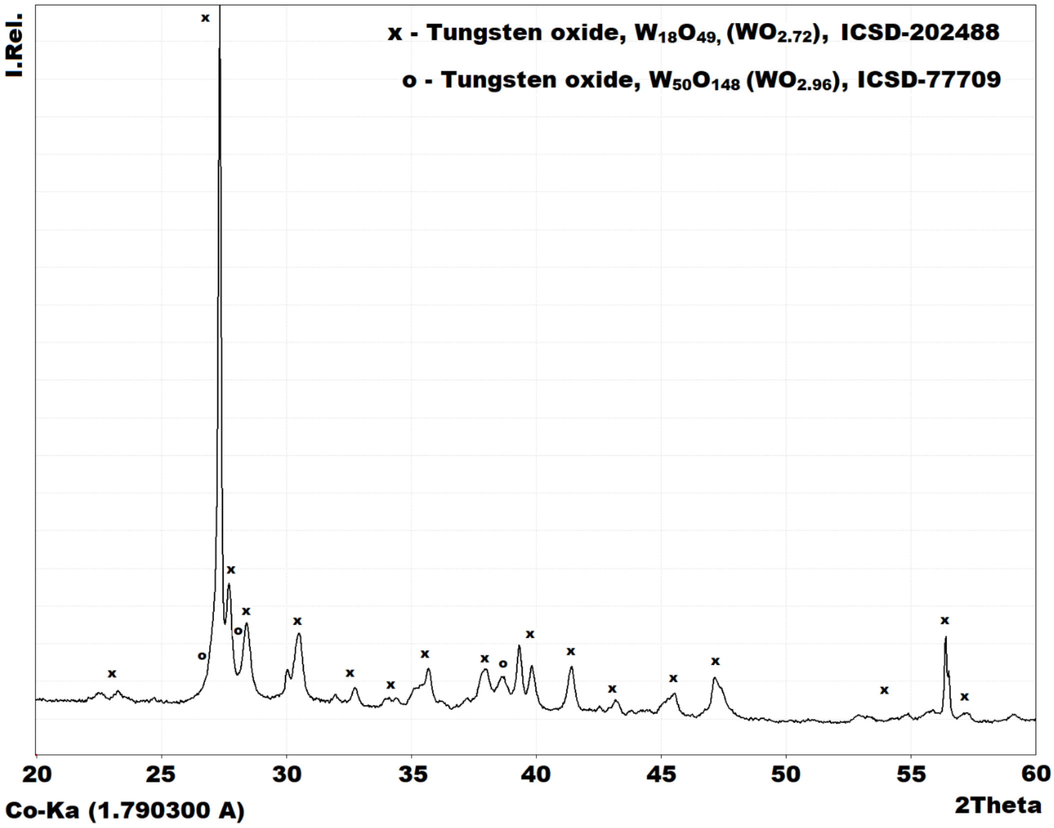

Figure S14. XRD diffractogram of the γ-WO_3/a-SiO_2 nanofibers reduced into $W_{18}O_{49}$/a-SiO_2 in a tube furnace under hydrogen atmosphere (100 Pa) by shock heating at 800 °C and 1 h dwell time. $W_{18}O_{49}$ (x) and $W_{50}O_{148}$ (o, minor phase) phases were detected.

STEM-HAADF/EDS Analysis of $W_{18}O_{49}$/a-SiO_2 Nanofiber

In order to understand the nanofiber/nanowhisker interface, a zone between nanofiber and two V-connected $W_{18}O_{49}$ nanowhiskers was analyzed via STEM-HAADF and STEM-EDS (see **Figure S15**). The analysis revealed the elemental prevalence of tungsten and silicon in the different parts of the interconnecting zone (**Figure S15 b** and **c**). The STEM-HAADF analysis (**Figure S15b**) shows clearly that the V-shaped nanowhiskers are very bright indicating that they are tungsten-rich. The STEM-EDS mapping of the tungsten, visualized as a blue area in **Figure S15c**, corresponded to the nanowhiskers, but the EDS signals of tungsten and silicon partially overlay - see area 1 in **Figure S15c** and the spectra in **Figure S15d**. Therefore, a specific site (area 2) was analyzed, where the amorphous silica was visibly separated from the tungsten oxide nanowhiskers (see the red area in **Figure S15c**). However, precise quantitative analysis of the nanofiber was challenging.

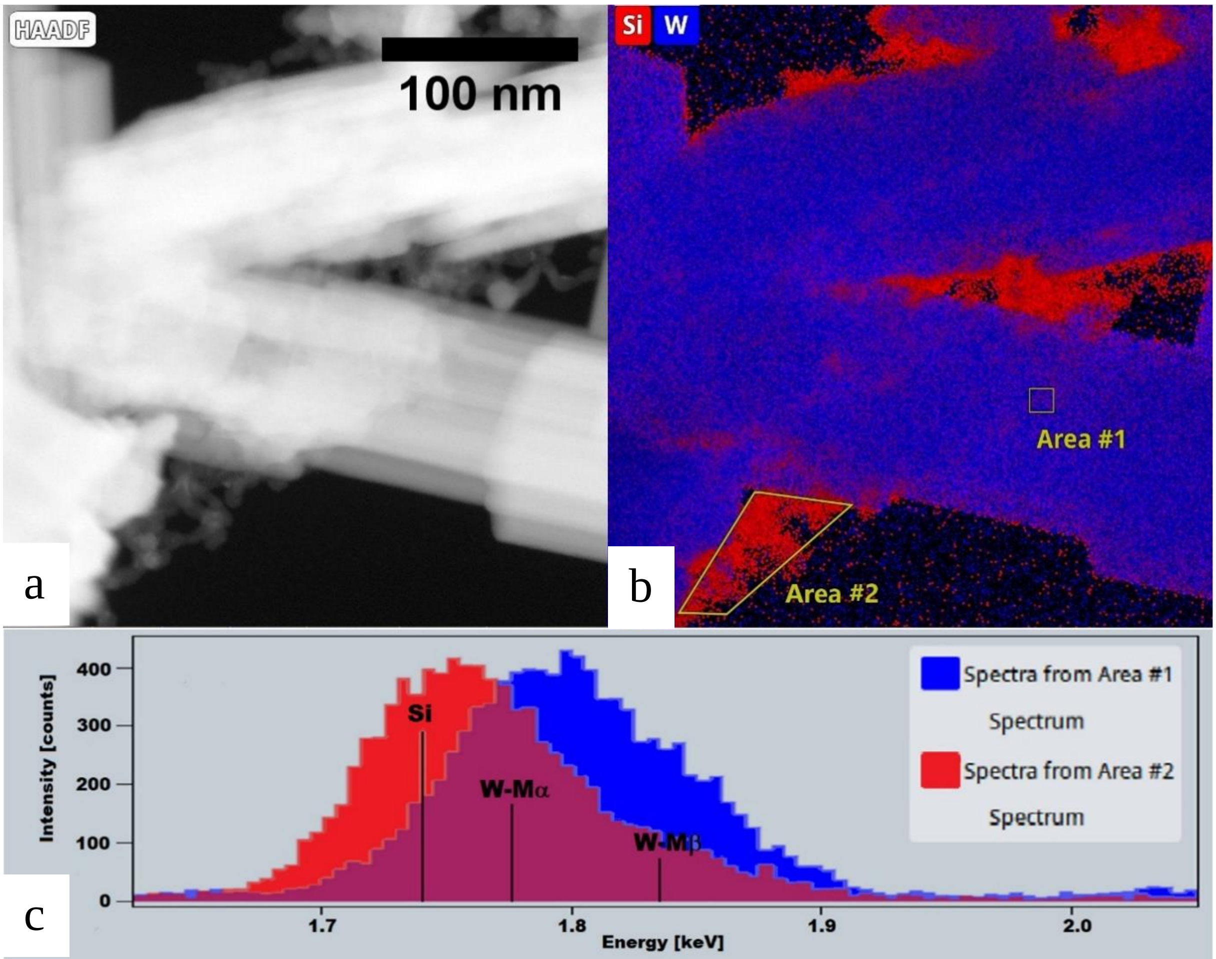

Figure S15. a) STEM-HAADF image of $W_{18}O_{49}$/*a*-SiO_2 nanofiber. b) STEM-EDS counterpart showing elemental mapping of tungsten (blue) and silicon (red). c) EDS energy spectrum showing the tungsten presence in Area #1 and #2 (b).

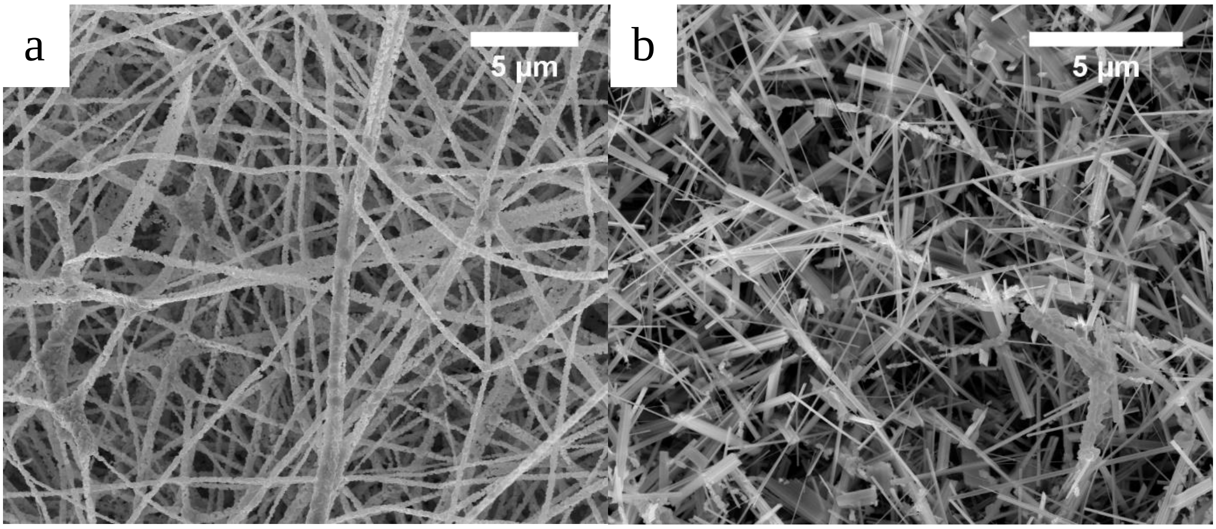

Figure S16. SEM images of γ-WO_3/*a*-SiO_2 nanofibers processed in the tube furnace under vacuum at a) 800 °C and b) 900 °C for one hour.

Evidently, 800 °C is not a sufficient temperature for the $W_{18}O_{49}$ nanowhisker growth (a), which corresponds closely with the *in-situ* observation in the μReactor within the SEM (see **Figure S9**, t = 0 s). On the other hand, the temperature of 900 °C (b) is inducing the growth of WO_{3-x} nanowhiskers.

However, the nanofibrous structure is not preserved and nanowhiskers with a broad distribution of length and thickness are formed. Interestingly, the reaction in a tube furnace at 900 °C under vacuum results in a similar morphology as in the μReactor counterpart (see **Figure S9ab**). Both experiments in the tube furnace (800 and 900 °C under vacuum) were performed as a direct comparison of tube furnace reactions with *in-situ* observed reactions in the μReactor. Both approaches are closely comparable. Therefore, the μReactor within SEM is highly suitable as a development tool.
